# Comparison of the conversion method with gravel unfolding to obtain dose values from photon spectra

**DOI:** 10.1093/rpd/ncad251

**Published:** 2023-09-21

**Authors:** Harald Dombrowski

**Affiliations:** Physikalisch-Technische Bundesanstalt, Bundesallee 100, 38116 Braunschweig, Germany

## Abstract

Modern gamma-ray spectrometers based on solid-state scintillators are increasingly being used in fields where previously dosemeters were applied. From the spectra, precise fluence and dose information can be derived. The most important methods of doing this are investigated in detail; the conversion method, which is based on weighing functions; and unfolding, here using the GRAVEL algorithm. Both methods can be used to process any kind of spectra, regardless of the type of spectrometer employed. The GRAVEL algorithm is described in detail. The implementation of both methods is shown and results of examples are compared. Advantages and disadvantages of both techniques are discussed. For the first time, most precise conversion data up to 20 MeV are published, even extending the quantity H^*^(10) to this energy. They may serve for the improvement and harmonisation of dose rate measurements using scintillation spectrometers, e.g. those used in early warning systems.

## Introduction

Compact scintillation detectors are being used increasingly in applications where they are replacing conventional dosemeters like Geiger–Muller counters, proportional counters or ionisation chambers^(1)^. They are, for instance, being installed in European early warning network systems. Because the same detector is used to record spectra and dose rate values, such instruments are also called spectro-dosemeters. Though such instruments have been available for several years, there is a lack of information on how to obtain precise dose rate information from spectra. This is because either the considered energy range of former publications is very limited or measured conversion coefficients had a high uncertainty. However, in all cases where intense neutron radiation is emitted, the detection of photon energies up to 20 MeV may be necessary. This work is based on precise Monte Carlo (MC) simulations, which are verified by measurements. Newly derived conversion coefficients with an uncertainty < 2% are provided in the annex for users of such instruments and scientists. Furthermore, the implementation and peculiarities of the important GRAVEL unfolding algorithm are described in detail. This algorithm is very suitable for highly resolved spectra.

In comparison with detectors based on NaI crystals, novel systems with LaBr_3_, CeBr_3_ or SrI_2_ crystals offer a much higher energy resolution, so that the spectra allow the better separation of gamma peaks. LaBr_3_ and CeBr_3_ crystals produce very fast light pulses, so that they may be used in time-critical applications like time-of-flight measurements at an accelerator. Handy and inexpensive digital pulse processing and read-out electronics have been developed in recent years, so that the prices of novel, uncooled detector systems can increasingly compete with those of conventional dosemeters. The advantages of spectro-dosemeters are the comparably high sensitivity, their compact size and their good commercial availability. The biggest advantage, however, is the availability of additional spectrometric information, e.g. on radionuclides. Furthermore, the performance of the dose or fluence calculation only depends on the applied software. This is a major difference in comparison with conventional dosemeters, which are mechanically built to display values in terms of one selected dosimetric quantity. Fluence measurements are not possible by using dosemeters if the spectrum is unknown.

The utilisation of spectro-dosemeters as dosimetric secondary standards is an outstanding area of application. By optimising software parameters, an almost ideal energy response can be realized. In metrology, spectro-dosemeters can be used to characterise standardised radiation fields. In the past, detectors with hyperpure germanium crystals were often used for such purposes. However, also the use of scintillation detectors may result in sufficiently precise fluence or dose data. In this paper, there will be some examples where spectra were recorded in photon fields created by radioactive sources or an accelerator.

## Material and methods

The investigated detector systems and the methods to derive (total) fluences and dose values from measured spectra will be described in the subsections below.

### Detector system

Two custom-made design scintillation detectors with photomultipliers were commissioned. One has a cylindrical CeBr_3_ crystal with a length of 1″ and a diameter of 1″, the other is identical in construction but the length and the diameter are both 1.5″. Commercial detector systems with crystals of such dimensions are very common, e.g. for environmental monitoring of ionising radiation. This crystal type was chosen because it has similar properties to LaBr_3_, which has become very popular in the last years. The content of radioisotopes of novel CeBr_3_ crystals is however extremely low, so that the inherent radioactivity is even hard to measure with a sensitive spectrometer. In contrast, LaBr_3_ spectra show patterns caused by inherent alpha, beta and gamma radiation, which is emitted by the isotopes ^138^La and ^227^Ac. The relative energy resolution of CeBr_3_ is slightly lower than that of LaBr_3_ (~4% in comparison with 3% at 662 keV, respectively), especially at low energies. However, the absolute energy resolution will be sufficient for many applications, as will be shown below. It is favourable to use a CeBr_3_ detector in neutron physics because CeBr_3_ has a rather low cross-section for neutron reactions. Also in this respect, Ce is favourable in comparison with La. Neutron reactions in the detector would create an unwanted background. In some accelerator experiments, neutrons are produced with a high fluence.

Both chosen detectors are equipped with a photomultiplier of the Hamamatsu 13 089 type because this multiplier is extremely fast and has a low jitter, which is a presupposition for the planned use in accelerator-based time-of-flight applications in the nanosecond range. The measured rise time of the pulses of the whole system is 8 ns. The efficiency of the bigger crystal corresponds to the efficiency a germanium crystal of 36% efficiency, as CeBr3 has a relatively high density of 5.2 g per cm^3^. Because the materials LaBr_3_ and SrI have quite similar properties, the results derived below can also be applied to detectors of the same dimensions based on the latter materials. The detector signals are read out by using the MPA-3 multi-parameter data acquisition system by FAST ComTec, which includes Silena ADCs.

### Photon fields

In this work, radioactive sources (^137^Cs, ^22^Na and ^207^Bi) are used to produce well-defined photon fields with emission energies up to 1.8 MeV. High-energy photons are produced at PTB’s ion accelerator facility (PIAF), which includes a direct current accelerator and a cyclotron.

### Methods to convert photon spectra to fluences or doses

The shape of a measured spectrometric pulse height distribution is governed by the efficiency of the utilised detector. The efficiency depends on (a) the mass-energy absorption coefficient of the active detector material, (b) the housing of the detector, which absorbs a fraction of the incoming photons and (c) the size of the active detector volume, e.g. the size of a scintillation crystal^(2)^. The mass-energy absorption coefficient both of the detector material and of the housing is a function of the energy of the incoming photons. The peak efficiency of the detector is dependent on the linear attenuation coefficient. Hence, a measured spectrum is detector-specific and has to be converted so that a result in terms of a well-defined quantity such as air fluence, air kerma or ambient dose equivalent, in short H^*^(10), is obtained. The quantity H^*^(10) plays a central role in radiation protection according to the European safety standards^(3)^, but it is also useful in the context of mixed radiation fields. This is because it allows a combined quantification of all kinds of ionising radiation at a certain location (by taking into account the biologic effectiveness towards human beings)^(4)^. The following methods have been used in order to do this:

- Unfolding using iterative algorithms or maximum likelihood estimation methods: The detector response matrix of a detector is calculated by using a MC code. The agreement of an estimate of the undisturbed fluence spectrum (which is multiplied by the response matrix) with the measured spectrum is optimised by a recursive method. The agreement may alternatively be optimised by minimising the entropy of the agreement between a reference spectrum and a solution spectrum. In this case, the solution spectrum is again derived iteratively from the measured spectrum in closed form in combination with estimated uncertainties, with the possibility to take into account an additional difference between predicted values and measured values. As an iterative method, GRAVEL will be used (mentioned later). An overview of unfolding techniques is found in^(5)^.
- Unfolding by matrix inversion: This is a theoretical method to solve the basic equation, which describes the relationship between fluence and measurement. Uncertainties are not taken into account in this approach. Under real conditions, this will not lead to a consistent solution, especially if the number of channels is high, because the uncertainties, even if they are very small, will ruin the results completely.
- Conversion of spectra: All channels of a measured spectrum are multiplied by conversion coefficients, which directly convert the spectrum into a fluence, air kerma or H^*^(10) spectrum. This method is very often used in commercial instruments, but was also successfully tested in scientific applications^(1)^. Details are explained later.
- Evaluation of photo peaks: If the energy resolution of a detector is so high that separate photo peaks are visible in a spectrum, the number of counts in a peak is proportional to an activity concentration or to specific activity of some radioactive material in the vicinity of the detector. A factor which converts counts in a peak into a physical quantity, like activity, is typically obtained from an MC simulation. It depends on the geometry of the radiation source, which has to be known or to be assumed. Based on geometrical assumptions, e.g. those of an activity distribution, a dose rate can be calculated. The disadvantage of the method is that a high but unknown uncertainty may result from the model-dependent geometrical assumptions. Furthermore, only physical processes that produce photo peaks can be taken into account.

In the following, the term of measured spectrum denotes a measured pulse height distribution (whose x-axis is proportional to the deposited energy), while the term (unfolded) spectrum denotes an energy distribution of the photon fluence, i.e. the spectral fluence.

### Monte Carlo simulations for spectrum conversion and unfolding

**Figure 1:**
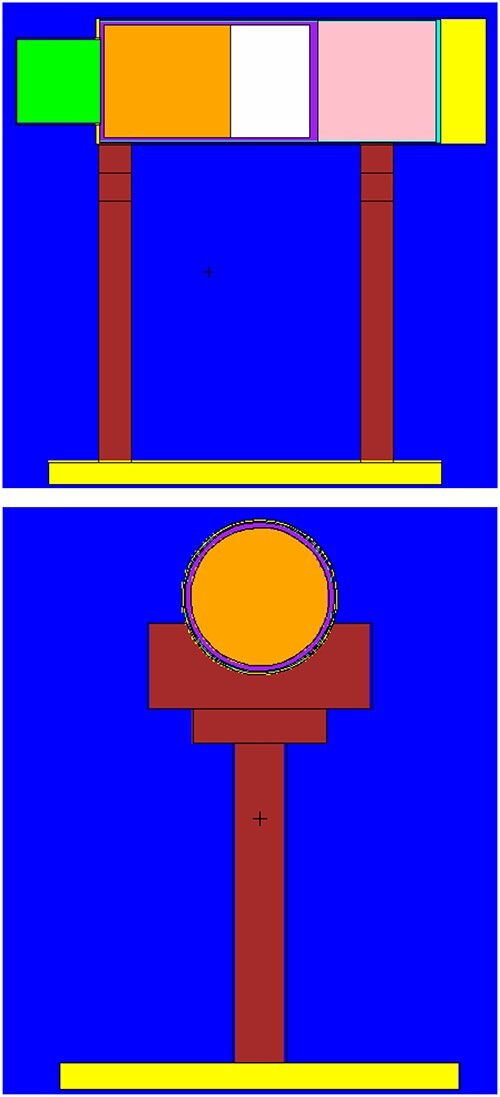
MCNP model of a scintillation detector with a 1.5″ × 1.5″ CeBr_3_ crystal (green). The different materials are displayed in false colours. The diverse components of the photomultiplier behind the crystal are represented by mean densities of the referring materials.

The geometries of two detectors with CeBr_3_ crystals of a different size were implemented in MCNP 6.2. The crystals have a cylindrical shape with (a) a length of 1″ and a diameter of 1″ or (b) a length of 1.5″ and a diameter of 1.5″. Three basic MC models of the detector were used as follows:

(A) a pure crystal with an aluminium encapsulation with a thickness of 0.5 or 5 mm;

(B) a crystal with an aluminium encapsulation with a thickness of 0.5 or 5 mm attached to a simple scattering body made of aluminium and steel, positioned directly behind the crystal;

(C) a sophisticated model of the complete detector and its holder with all known components (including, for example, the Teflon wrapping of the crystal and the Mu-metal shield of the photomultiplier), as displayed in Figure 1 (very thin layers are not visible on this scale). The housing of the front and the sides of the crystal consists of aluminium with a thickness of 0.5 or 5 mm. Both cases were taken into account.

The thin aluminium housing corresponds to the actual factory-provided design, while the thick housing may simulate additional absorbers (also made of other materials like polyethylene), or an additional housing around the detector, e.g. for weather protection, as a simplified approximation.

In MCNP, there are pre-defined methods, so-called tallies, which define the way of spectrum summing and the units of the spectrum bins. Here, the tally F8, pulse height distribution in a cell, was selected in the input file, whereby the cell is identical with the CeBr_3_ crystal (bin unit: MeV). Using the default settings concerning transport parameters, all physical effects were taken into account, which are relevant for photon, electron and positron transport. These include the generation of bremsstrahlung, coherent scattering, the generation of X-rays and fluorescence (lower photon and electron energy cut-off: 1 keV). As an exception, the effect of Doppler broadening is switched off in MCNPX by default, but not in the other MCNP versions.

#### Comparison of Monte Carlo codes

Model (A) was used to compare different MC codes, because the simplicity of the model is very good at revealing differences which are merely caused by the MC code. Simulated spectra using MCNP 5, MCNP X, MCNP 6.2 and Penelope^(6)^ were calculated for comparison and for the purpose of gaining some information about typical uncertainties. The codes were used with standard settings concerning the physics model, for example, photon and electron transport was switched on. Furthermore, the newest libraries were used in all simulations. The MC codes calculate uncertainties for each bin of a simulated spectrum, but some systematic uncertainties are not included in these numbers. Two examples are shown: A point source, which is located 1 m away from the front of the crystal and which is positioned on the symmetry axis of the crystal, irradiates this crystal with mono-energetic photons. In the first example, the size of the crystal is 1″ × 1″ and the photon energy is 660 keV. In the second example, a 1.5″ × 1.5″ crystal is irradiated with 10 MeV photons. The wall thickness of the aluminium casing is 5 mm in both cases.

**Figure 2:**
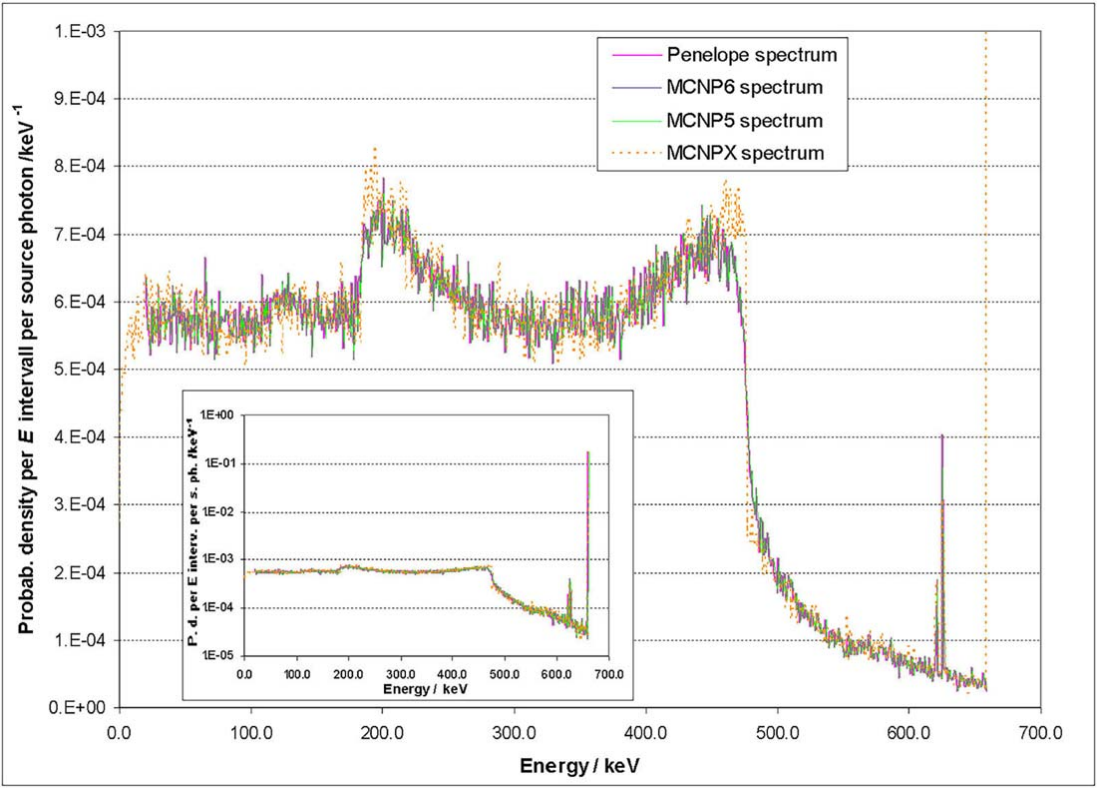
Simulated spectrum of the energy deposition of mono-energetic 660 keV photons in a 1″ × 1″ CeBr_3_ crystal wrapped in aluminium of 5 mm thickness (Penelope data by^(6)^).

**Figure 3:**
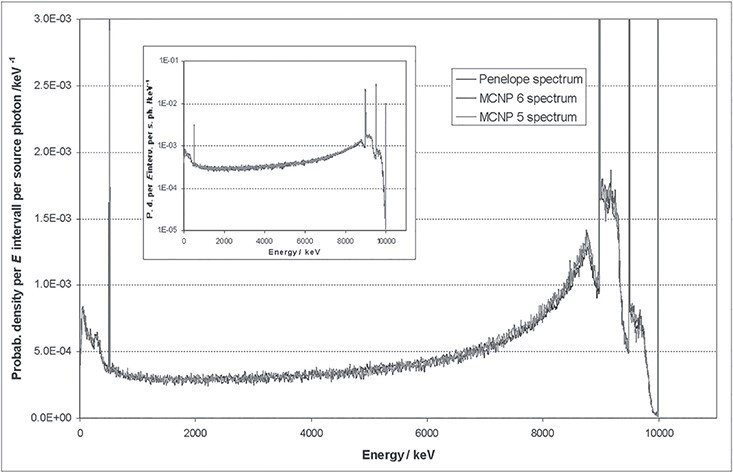
Simulated spectrum of the energy deposition of mono-energetic 10 MeV photons in a 1.5″ × 1.5″ CeBr_3_ crystal wrapped in aluminium of 5 mm thickness (Penelope data by^(6)^).

Figures 2 and 3 illustrate that there is a very good agreement between the results of the different codes. A more detailed evaluation of the full-energy photo peaks exhibits differences which are <3% in all studied cases. The statistics of the simulations were so good that observed deviations have to be attributed to systematic uncertainties in physics models and libraries. Calculated peak efficiencies are important for the implementation of the conversion method but also have an influence in unfolded results. A closer look reveals that the spectrum calculated with MCNPX shows very sharp Compton edges which are unphysical, because the electron transport is simplified when standard settings are chosen. For the calculation of detector response matrices, model (C) implemented in MCNP 6.2 was used.

#### Folding of Monte Carlo spectra with the detector resolution

**Figure 4:**
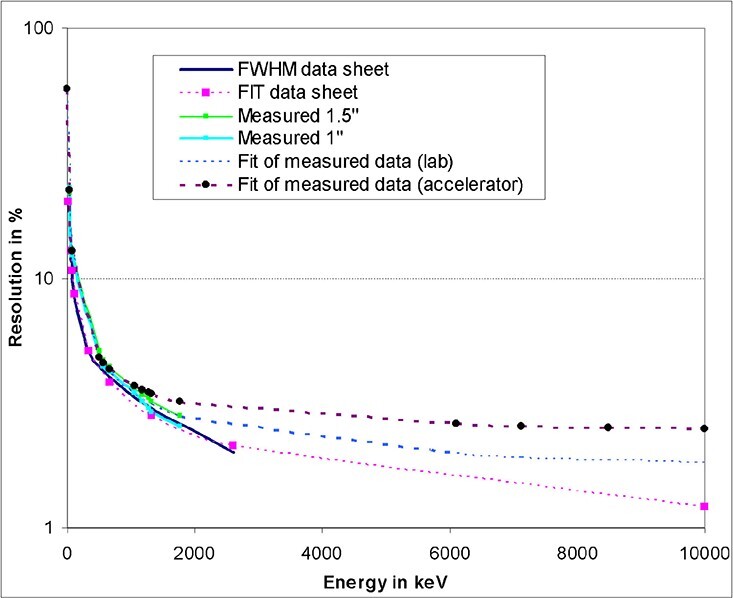
Energy resolution of two CeBr_3_ detectors of different sizes. Different data sets are compared.


A presupposition of a comparison of simulated spectra with measured spectra is that the detector resolution is known. Figure 4 shows the energy resolution of two detectors which are equipped with CeBr_3_ crystals of different dimensions: 1″ × 1″ and 1.5″ × 1.5″. The measured energy resolutions are very similar. The fact that the smaller crystal has a slightly better energy resolution was neglected, by fitting one common resolution curve. Measured data exist up to 7.1 MeV. The fitted curve was extrapolated to 10 MeV. This is possible because the relative energy response at high energies is almost constant. The fitted curve was checked by applying fits to spectra recorded in the R-F photon field (defined by ISO) at the accelerator facility at PTB (see below). The numerical value equation

(2.1) \begin{equation*} R(E)=\sqrt{a^2+{b}^2/E+{c}^2/{E}^2} \end{equation*}

is used to fit the energy resolution. Because measurements at the accelerator are influenced by a higher level of electronic noise in comparison with pure laboratory measurements, the fit of the detector resolution had to be shifted up to higher values, especially at higher energies. The parameters of the respective *R*(*E*) function are: a = 2.3094666, b = 92.055951 and c = 489.66835, whereby *E* is the dimensionless value of the energy in keV. The result is the detector energy resolution in percent.

**Figure 5:**
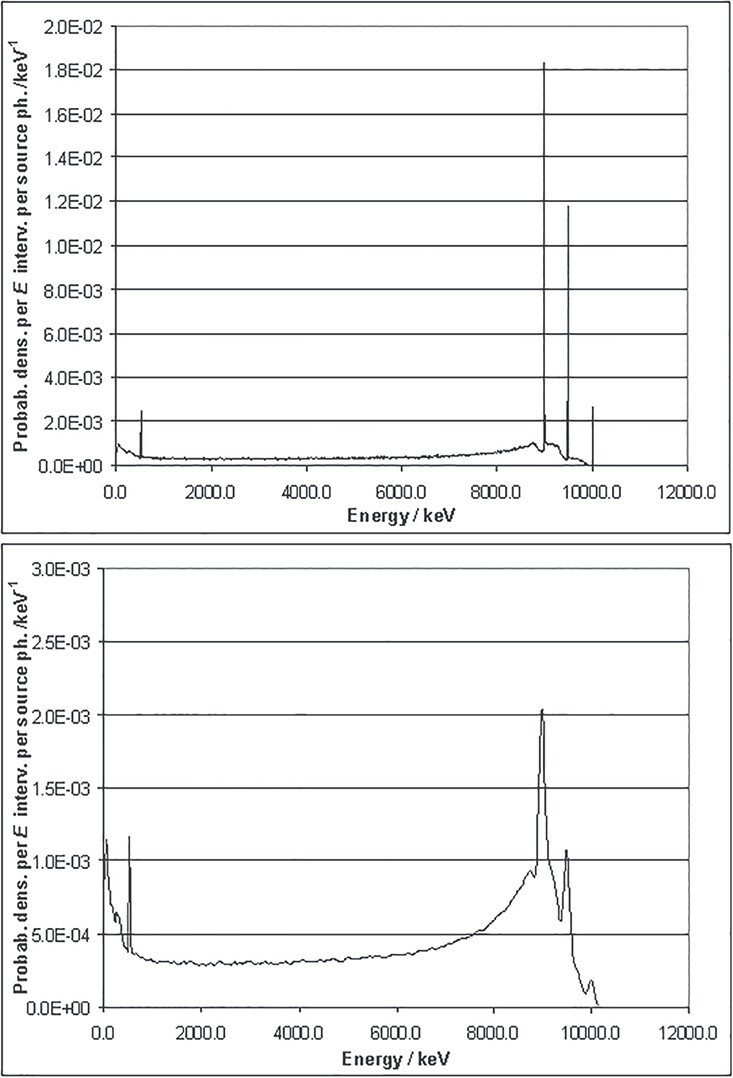
MCNP energy spectrum of 10 MeV photons deposited in a 1″ × 1″ CeBr_3_ detector (top) and the same spectrum folded with the detector resolution (bottom). The escape peaks at 9.0 and 9.5 MeV are more pronounced than the photo peak at 10.0 MeV.

The MC spectra have to be folded with the energy resolution to obtain spectra which resemble measured spectra. The formula used to calculate each channel of the folded spectrum is:


(2.2) \begin{equation*} {N}_j^{new}=\sum \limits_k{N}_k\cdot \frac{1}{\sqrt{2\pi}\sigma}\cdot{e}^{-\frac{1}{2}\cdot{\left(\frac{E_j-{E}_k}{\sigma}\right)}^2}\cdot \varDelta E \end{equation*}

with


$$ \sigma =\frac{R(E)}{2.3548}, $$

where *R*(*E*) denotes the full width half at maximum of the peak, *N_k_* the counts of one channel of the MC spectrum and *E_j_* the energy of channel *j*. As an example, Figure 5 shows a pure MC spectrum and a spectrum folded with the detector resolution.

#### Verification of Monte Carlo calculations using radioactive sources

The measured spectra of radioactive sources are compared with simulated spectra to check the correctness of the MC results, i.e. to verify that the geometries and materials were implemented correctly in the MCNP code. The activity of each source is known with an uncertainty of 1%. This will allow a quantitative verification of the analysis methods for the quantitative evaluation of spectra described below. A presupposition of such a comparison of measured and simulated curves is that the detector resolution is known. Examples are shown in the figures mentioned later. The whole installation is built up on an aluminium bar raised by a stand in the middle of a laboratory room. Furthermore, it is built in such a way that as little as possible material is present in the vicinity of the installed source and the detector (both held on the bar by lightweight spacers). Scattering is avoided to the best degree possible, because the simulation of complex surroundings is not possible with a reasonable effort.

Three sources, a ^137^Cs, a ^22^Na and a ^207^Bi source, are positioned at a distance of 39 cm from the detector entrance window (0.5 mm Al) at one time. Because the sources are only covered by a thin plastic foil, electrons as well as positrons can escape from the sources and reach the detector in addition to the photons, so that they also contribute to the total dose. ^207^Bi emits gamma lines at 73, 75 and 85 keV (three unresolved X-ray lines), and 570, 1064 and 1770 keV. The ^22^Na source emits photons at 511 and 1275 keV. The ^137^Cs releases photons at 32 keV (two unresolved X-ray lines) and at 662 keV. Depending on the noise threshold of the ADC, the lower limit of the measured spectra lies at ~20 keV. In spite of the calculation of net spectra from shadow cone measurements, there are (few) events from scattering in the room. To account for this in a very simplified way, the simulations include the source and the detector in an air barrel with a boundary layer of dense air, so that some diffuse scattering is possible.

**Figure 6:**
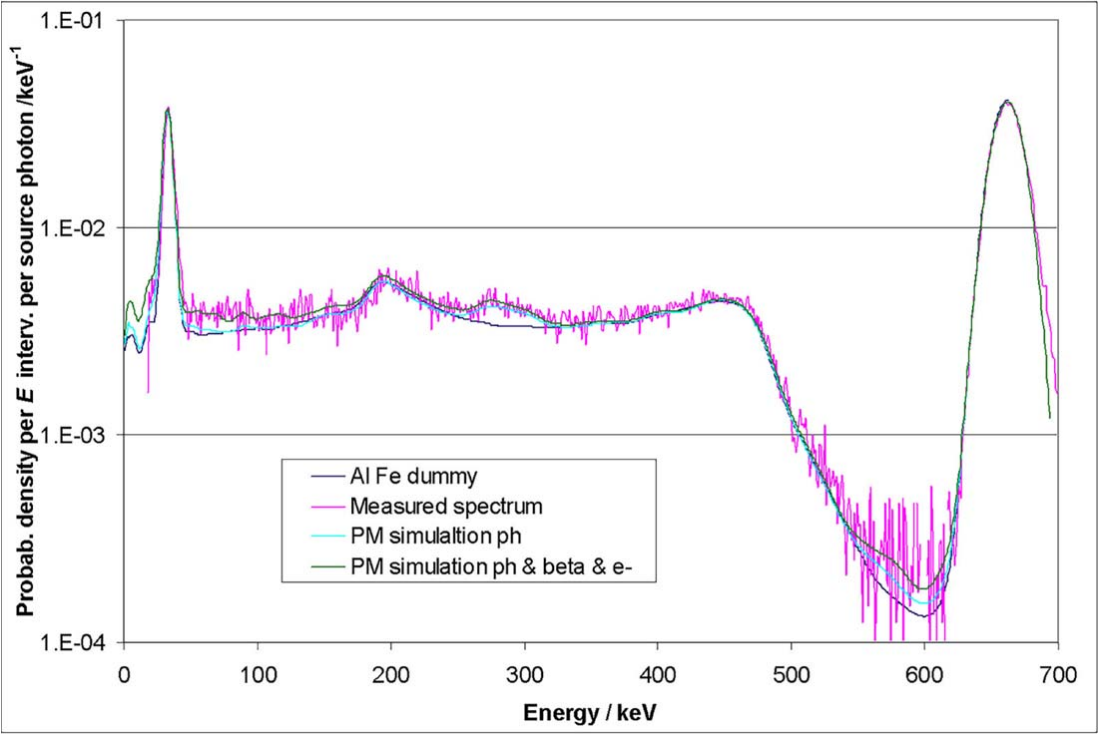
Comparison of measured and simulated ^137^Cs spectra of a 1.5″ × 1.5″ CeBr_3_ detector.

**Figure 7:**
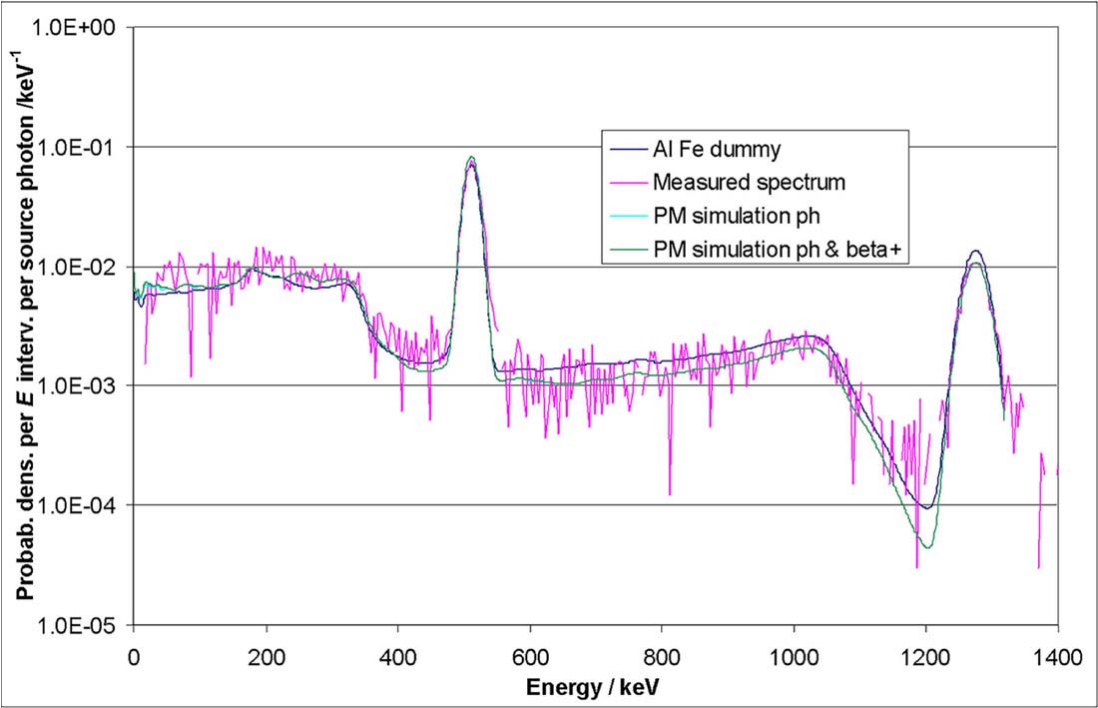
Comparison of measured and simulated ^22^Na spectra of a 1.5″ × 1.5″ CeBr_3_ detector.

**Figure 8:**
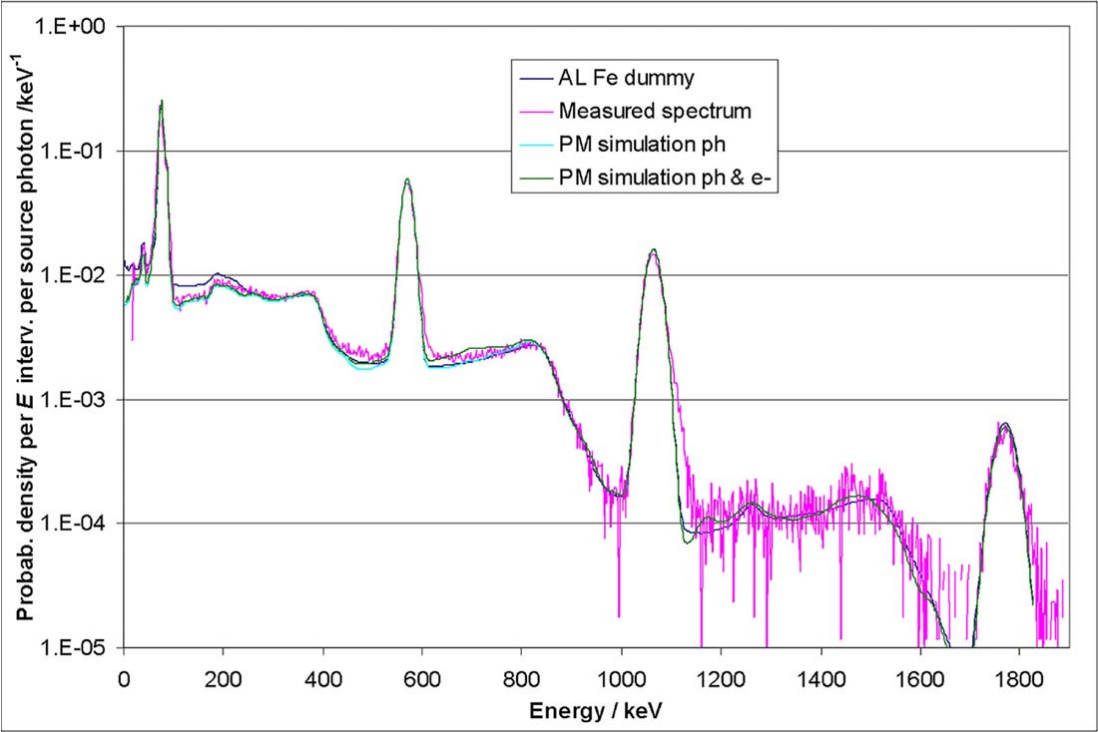
Comparison of measured and simulated ^207^Bi spectra of a 1.5″ × 1.5″ CeBr_3_ detector.

The MC simulations with a very simple geometry (dark blue curves) already follow the measured data (magenta curves) quite well. The complete MC model leads to an even better agreement (light blue curves). Because open sources were used, the influence of the emitted electrons and positrons was also included in the simulations (green curves). The latter curves show the best agreement, even in detail. For example, the background level next to the 32 keV peak in the ^137^Cs spectrum is best reproduced by the green curve of the full simulation. However, for most applications, especially if the calculation of total fluences or doses is intended, the simple approach using a wrapped crystal and a dummy behind (to simulate backscattering) will lead to sufficient results. The measured spectra in Figures 6–8 are normalised relatively to the MCNP spectra by taking into account the actual source activities and the total emission probabilities as well as the different energy bin widths.

The agreement of measured and simulated spectra is good, which serves as a verification of the correct implementation of geometries and materials in MCNP code. The code is able to transport photons and electrons or positrons, as well. This will be quantitatively shown below, where spectra of ^137^Cs, ^22^Na and ^207^Bi sources will be evaluated quantitatively using the known activity of the sources. The correctness of the geometrical dimensions was checked by using the graphical output of MCNP (examples are shown in Figure 1).

### The conversion method

This method is widely used because it allows the swift conversion of measured spectra into dose rates in fractions of seconds. The idea is to multiply all measured counts of each channel of the spectrum by an energy-dependent coefficient, which includes all information on the detector response^(2)^. The integral of the obtained fluence or dose spectrum is the total fluence or dose, respectively. However, the fluence or dose spectrum is only calculated as an intermediate step. It does not represent the actual, undisturbed fluence or dose spectrum at the position of the detector. The latter are only obtained by unfolding (section given later). An important prerequisite for a successful application of this method in unknown fields (where the direction of the impinging photons is not known) is that the angular response of the detector is isotropic. This applies in the case that there is no directional information of the incoming photon fluence available. Here, the response matrices have been calculated for a photon beam hitting the detector under 0°, in the direction of the central symmetry axis of the cylindrical detector crystal. However, the isotropic detector response also allows the quantification of the background radiation, which is produced by photons and other ionising radiation of undefined origin (scattered photons, radioactivity, secondary cosmic radiation, etc.), coming from all possible directions.

#### Short description of this method

First of all, continuous spectra are transferred to counts in energy bins with a defined energy width (the typical task of the multiple channel analyser of an instrument). The basic equation which has to be solved if a spectrum is to be converted to a fluence or dose is $\mathop{\mathrm{N}}\limits^{\to } = \mathbf{M}$ • $\mathop{\mathrm{G}}\limits^{\to }$. This algebraic matrix equation expresses that the measured spectrum $\mathop{\mathrm{N}}\limits^{\to }$ results from the actual fluence in nature $\mathop{\mathrm{G}}\limits^{\to }$ by a multiplication of the vector $\mathop{\mathrm{G}}\limits^{\to }$ with the response matrix of the detector **M**, whereby all matrix elements have to be adjusted to the chosen energy scales of the vector (or vice versa). The matrix is quadratic if the energy scales of both vectors are identical. A dosimetric quantity, e.g. the ambient dose equivalent H^*^(10), is obtained by the scalar product of the fluence in nature $\mathop{\mathrm{G}}\limits^{\to }$ with $\mathop{{\mathrm{G}}_{\mathrm{H}}}\limits^{\to }$, the fluence to dose conversion vector: H^*^(10) = $\mathop{{\mathrm{C}}_{\mathrm{H}}}\limits^{\to }$ • $\mathop{\mathrm{G}}\limits^{\to }$. When both equations are combined, one obtains H^*^(10) = ($\mathop{{\mathrm{C}}_{\mathrm{H}}}\limits^{\to }$ • $\mathbf{M}$^−1^) • $\mathop{\mathrm{N}}\limits^{\to }$ =: $\mathop{\mathrm{V}}\limits^{\to }$ • $\mathop{\mathrm{N}}\limits^{\to }$ (the matrix inversion requires a quadratic matrix). The thus obtained scalar vector product can be re-written in the form


(2.3) \begin{equation*} {H}^{\ast }(10)={w}_1\times{E}_1\times{n}_1+{w}_2\times{E}_2\times{n}_2+\dots{w}_Z\times{E}_Z\times{n}_Z \end{equation*}

when the number of energy bins is *Z*. The mean energy *E*_i_ of each energy bin is extracted because, as a first approach, the resulting dose is proportional to the photon energy. Any other physical quantity, e.g. air kerma, is obtained analogously by applying a different conversion vector, which is related to this quantity. The existence of the coefficients cannot strictly be proven mathematically. However, it was empirically shown in many publications that this approach works, e.g.^(7–9)^. The determination of the coefficients *w_i_* can be done by defining broader energy ranges, first of all, for which one mean conversion factor is valid. The coefficients were calculated sequentially proceeding from the lowest range to the highest range. The dose of the reference spectrum must be known from measurements (experimental approach) or from the calculation of the simulated input fluence (MC approach). In this work, MCNP simulations of mono-energetic photons were used because the wide energy range needed in this work cannot be covered by measurements with radioactive sources.

At an energy >1 MeV, the cross-section of pair production gradually rises. Hence, at higher energies, more and more 511 keV photons leave the detector without an interaction. This produces two escape peaks in addition to the photo peaks of the incoming photons. At some MeV (and above) the escape peaks will be larger than the photo peak (Figure 5). When the coefficients *w*_i_ are calculated in such energy regions, it is important that every energy region covers the referring photo peak and both escape peaks. Otherwise, alternating negative and positive conversion coefficients will be found, which are unphysical.

If a thus obtained data set of coefficients is used in applications for calculated doses (or fluences), the results may show unsatisfactory effects. On the one hand, deviations of already determined coefficients from true values will influence the determination of other coefficients, which will be accordingly low or high. This effect is visible as an oscillation of the determined curve around the smooth true curve. On the other hand, the conversion will be imprecise if a peak is located close to the edge of an energy region, because the conversion factor is primarily valid for the middle of the region (or the energy where most counts of the reference spectrum entered).

**Figure 9:**
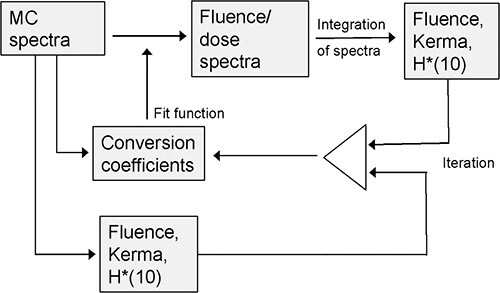
Scheme of the procedure to find conversion factors.

Both problems can be overcome by using splines of the determined coefficients, so that a different conversion coefficient is applied to each bin of the measured spectrum. The splines are fitted by polynomials resulting in analytic functions, which can be used easily in computer codes. The sets of coefficients (and their fit functions) are adjusted iteratively so that the total dose result of the whole spectrum is in agreement with the known reference dose values (Figure 9). Typically, after three to six iterations, the reference dose values of all energy regions are reproduced by the conversion curve within an uncertainty of 2%. This is approximately the uncertainty of the number of counts of the photo peaks in the simulated MC spectra. The selected energy regions and their representative energies as well as the obtained conversion coefficients are listed in the annex.

#### Calculation of H^*^(10) up to 20 MeV

ICRP (and ICRU) have published conversion coefficients, which are used to calculate H^*^(10) from fluence to air kerma in the energy range from 10 to 10 MeV^(10)^. The needed conversion coefficients above 10 MeV were calculated in this work, as detailed below.

Both air kerma *K*_a_ and collision kerma *K*_a,col_ are proportional to the energy fluence *ψ* = *E ·* Φ, the product of the energy and the particle fluence. To obtain the air kerma, the mass energy transfer coefficient, *μ_tr_/ρ* of (dry) air has to be known, whereas the mass energy absorption coefficient, *μ_en_/ρ,* enters the collision kerma calculation:


(2.4) \begin{equation*} {K}_a=\frac{\mu_{tr}}{\rho}\cdot \psi\ \text{and}\ {K}_{a, col}=\frac{\mu_{en}}{\rho}\cdot \psi \end{equation*}

The mass-energy absorption coefficient of dry air is tabulated^(11)^. The mass-energy transfer coefficient can be calculated by using the following equation when the *g*-function is known:


(2.5) \begin{equation*} \frac{\mu_{en}}{\rho }=\left(1-g\right)\cdot \frac{\mu_{tr}}{\rho } \end{equation*}

**Figure 10:**
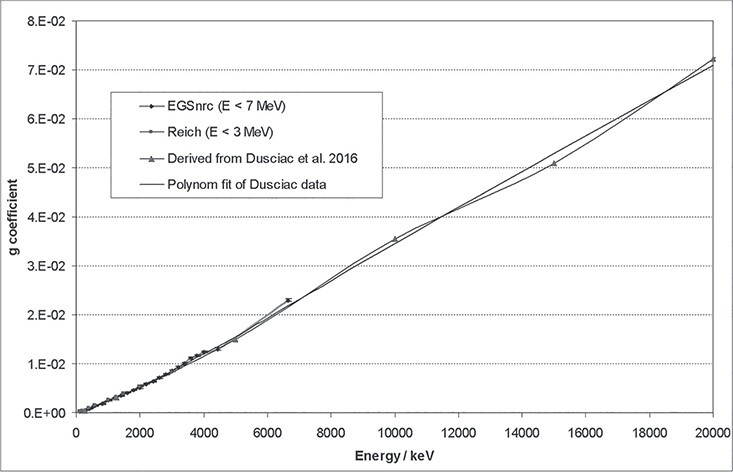
Data and polynomial fit functions of the g coefficient. The grey fit function (up to 20 MeV) is a polynomial of the 4^th^ degree, y(x) = bx + cx^2^ + dx^3^ + ex^4^, with b = 2.29968 ·10^−6^, c = 2.2162903 ·10^−10^, d = 1.3326679 ·10^−14^ and e = 2.6805943 ·10^−19^.

The coefficient g describes the average fraction of the kinetic energy of secondary charged particles which is lost in radiative energy-loss processes in the substance of air (here: preferably losses by bremsstrahlung). The effect is especially relevant at higher energies of some MeV. The *g*-function (Figure 10) was derived from a publication, in which it is indirectly included^(12)^. Thus, the air kerma and the ambient dose equivalent, H^*^(10), are calculated according to


(2.6) \begin{equation*} {K}_a=\frac{\mu_{en}}{\rho}\cdot \frac{1}{\left(1-g\right)}\psi; {H}^{\ast }(10)={h}_K^{\ast }(10)\cdot{K}_a \end{equation*}

**Figure 11:**
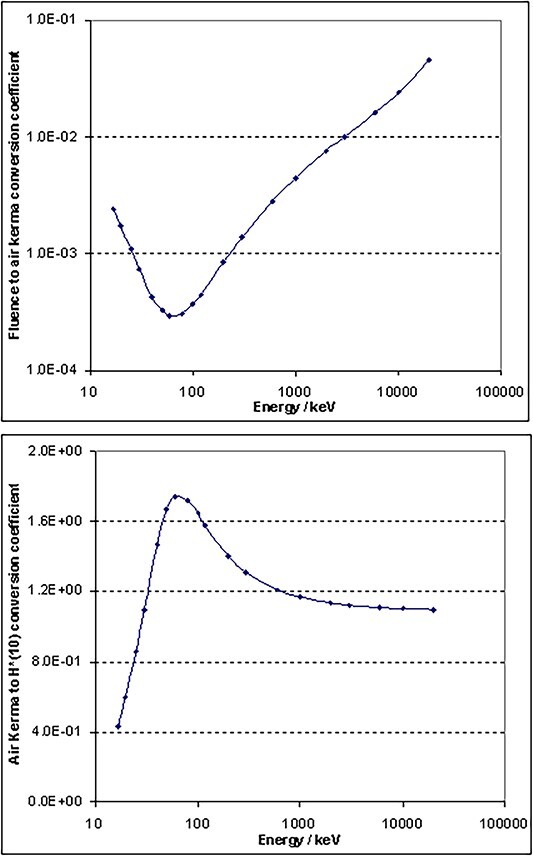
Fluence to air kerma conversion coefficient (top), related to a fluence of 1 cm^−2^ and air kerma to H^*^(10) conversion coefficient in Sv/Gy (bottom), using polynomial fit functions of this work in both cases.

A fitted curve of the coefficient ${h}_K^{\ast }$, which converts *K_a_* to H^*^(10), was published by Wagner et al. in 1985^(13)^ and can be found in the report. This function can be used up to 20 MeV. Thus, all needed ingredients are available to calculate H^*^(10) up to 20 MeV. In the cited research article^(12)^, the observation is expressed that ICRU and ICRP have actually published conversion coefficients from fluence to collision kerma, not to air kerma, even though air kerma is mentioned (wrongly). The conversion coefficients are plotted in Figure 11.

#### Calculated spectrum conversion coefficients

After verifying the correct implementation of the detector properties in MCNP, a simplified model (Model A defined earlier) was used to simulate the detector response of both CeBr_3_ detectors described above (crystal sizes 1″ × 1″ and 1.5″ × 1.5″). A point source at a distance of 1 m from the front of the crystal is assumed. The crystal is either wrapped by an aluminium layer of 0.5 or 5 mm, so that in total four configurations are investigated. The energy deposition of mono-energetic photons in the detector crystals was simulated by using the Penelope and MCNP codes. All energies, for which supporting points of the conversion curve are calculated, are listed in Table 8. This table also includes some other information which is relevant to calculate reference dose values. Small differences between the results of the codes (<2%) are due to uncertainties in the simulations. The photon transport is done in vacuum, so that the absorption of the air is neglected. This is possible because low energies (*E* < 30 keV) are of little interest.

The lower threshold of measured spectra, as shown in Figure 6, is typically above this limit in this work. A simplified geometry is used in this case, because the development of general conversion curves is intended, which can be used for any detector with a LaBr_3_, CeBr_3_ or SiI_2_ crystal of the dimensions 1″ × 1″ or 1.5″ × 1.5″. This simple model can be applied generally with good precision, because a sophisticated adoption of simulated spectra to measured spectra is not needed if only total fluences or doses are of interest. In such a case, the small differences between the simple model and the detailed model shown in Figure 3 are negligible. The conversion curves can be used for LaBr_3_, CeBr_3_ or SrI_2_ detectors, because the mass energy absorption coefficient and the density of these materials, which both govern the energy deposition, are very similar. This fact was experimentally confirmed in^(14)^.

This outcome is physically expected from the energy-dependent curve of the mass energy absorption coefficient (from low to high energies it decreases by some orders of magnitude), while the limited size of the crystal has an additional influence. At low energies, all photons are stopped in a thin front layer of the crystal, while, at very high energies, an increasing fraction of the photons will travel through the whole crystal without an interaction. At the low end of the energy scale, the thickness of the casing governs the amount of photons which reach the detector crystal at all. Therefore, the air kerma and H^*^(10) conversion curves (Figures 21 and 22) are practically identical at energies >100 keV. This is the first publication in which conversion curves up to 20 MeV are derived. It is interesting to see that the spectrum to fluence curves fall smoothly at high energies, which just looks like an extrapolation, while the air kerma and H^*^(10) curves rise again at the highest energies.

### Unfolding

In contrast to the conversion method, the idea of this method is to reconstruct the original fluence spectrum at the location of the detector. Afterwards, this spectrum can be converted to a dose spectrum. Total fluences and doses can be calculated from the fluence and dose spectra, respectively. As a prerequisite, the response of the detector to photons has to be well known in the energy range of interest. This means, that the vector $\overrightarrow{\mathrm{G}}$ has to be extracted from the equation $\overrightarrow{\mathrm{N}}$ = **M** • $\overrightarrow{\mathrm{G}}$, assuming that the measured spectrum $\overrightarrow{\mathrm{N}}$ and the detector response matrix **M** are known.

#### Short description of the GRAVEL algorithm

Because the unfolding of high-resolution spectra is necessary in this work, the iterative algorithm GRAVEL was chosen. It is part of the free UMG software package distributed by PTB^(15)^. This algorithm is based on the SAND II algorithm^(16)^, which was already published in 1967 and is still in use to date. GRAVEL was deducted from SAND II by including uncertainties. The basic formulas for iterations according to GRAVEL are:


(2.7) \begin{align*} {f}_i^{J+1}={f}_i^J\cdot \exp \left\{\frac{\sum \limits_k{W}_{ik}^J\cdot \log \frac{N_k}{\underset{i^{\prime}}{\varSigma }{R}_{ki^{\prime}}{f}_{i^{\prime}}^J}}{\underset{k}{\varSigma }{W}_{ik}^J}\right\} \nonumber\\<=>{f}_i^{J+1}={f}_i^J\cdot \exp \left\{\frac{\sum \limits_k{W}_{ik}^J\cdot \log \frac{N_k}{T_k^J}}{\underset{k}{\varSigma }{W}_{ik}^J}\right\} \end{align*}

where $k\in \left\{1,2,3,...,m\right\}$, $i\in \left\{1,2,3,...,n\right\}$ and $m\ge n$, with ${W}_{ik}^J=\frac{R_{ki}{f}_i^J}{\underset{i^{\prime}}{\varSigma }{R}_{ki^{\prime}}{f}_{i^{\prime}}^J}\left[\frac{N_k^2}{\sigma_k^2}\right]$ <=> ${W}_{ik}^J=\frac{T_{ki}^J}{T_k^J}\left[\frac{N_k^2}{\sigma_k^2}\right]$.

using the definition of the so-called test spectrum:


(2.8) \begin{align*} {T}_k^J=\sum \limits_i{R}_{ki}{f}_i^J\ and\ {T}_{ki}^J={R}_{ki}{f}_i^J \end{align*}

The meaning of the variables is the following (*element* denotes the number of counts in one bin):

**Table:** 

${f}_i^J$ :	i^th^ element of the unfolded spectrum of iteration *J*
${f}_i^1$ :	i^th^ element of the 1^st^ estimate of unfolded spectrum
${T}_k^J$ :	k^th^ element of test spectrum
${N}_k$ :	element *k* of measured spectrum
${\sigma}_k$ :	estimate of measurement uncertainty of bin k
${R}_{ki}$ :	response matrix element
${W}_{ik}^J$ :	normalisation matrix element
*J*:	iteration step (starting at 1)

By applying the GRAVEL (or SAND II) formulas, the test spectrum or refolded spectrum $\overrightarrow{\mathrm{T}}$, which is the unfolded spectrum *f* times the response matrix **R**, is iteratively adapted to the measured spectrum $\overrightarrow{\mathrm{N}}$. If the test spectrum and the measured spectrum agree, the logarithm in the iterative formula *f_i_* is zero, so that the whole exponent is zero. In this ideal case, the unfolded spectrum is not changed any more. The iteration has come to an end. The normalisation matrix **W** is calculated from the response matrix **R**. The scalar product of the columns of the response matrix times the unfolded spectrum is normalised to unity.

As a consequence of the construction of the GRAVEL unfolding formulas, spectrum bins with a high number of events *N_k_* (measured) and *f_i_* (unfolded) will be altered strongly, while spectrum bins with a few counts will barely be changed. The spectrum shape of bins with few counts will, therefore, be dominated by the first estimate of the unfolded spectrum. According to the formulas above, the unfolding of channels or matrix elements which are equal to zero does not work and has to be skipped by any unfolding code. If regions of adjacent bins contain zeros, which may especially be observed in the lowest or highest bins of a measured spectrum, artefacts may be a consequence in the neighbouring non-zero regions. This problem is overcome by excluding such zero bin regions from unfolding. Furthermore, all bins of spectra to be unfolded as well as all elements of the response matrix have to be positive. Otherwise, non-physical results would be obtained.

To start the iteration process, a first estimate of the unfolded spectrum is needed. If the measured spectrum shows clear photo peaks, it can be readily used as a first estimate. Alternatively, an artificial flat spectrum can be used on which the expected peaks are superimposed. There is no clear criterion after how many steps the iterative unfolding process has to be stopped. Because of the uncertainties in the measured spectrum, the iteration could be continued until infinity (a perfect agreement of the measured spectrum and the test spectrum is never reached). Therefore, a criterion for the optimum number of iterations is needed. One valuable indicator of each iteration *J* is the χ*^J^* parameter, which is defined as


(2.9) \begin{equation*} {\left({\mathrm{\chi}}^J\right)}^2=\sum \limits_k{\left(\frac{N_k-\sum \limits_i{R}_{ki}{f}_i^J}{\sigma \cdot \sqrt{F}}\right)}^2=\sum \limits_k{\left(\frac{N_k-{T}_k^J}{\sigma \cdot \sqrt{F}}\right)}^2 \end{equation*}

The parameter *F* denotes the degrees of freedom that are identical with the number of spectrum bins that are unfolded. In many cases, χ*^J^* reaches a minimum, or it falls below a typical value. A second indicator is the integral over the whole unfolded spectrum, i.e. the total number of counts of this spectrum. This indicator may also pass a minimum. The reason for this is the fact that after a number of iterations, the unfolded spectrum will start to oscillate because of the misinterpretation of structures just caused by statistical uncertainties. The oscillations show peaks and valleys with the width of the detector resolution. This effect is caused by the widths of peak structures in the response matrix. When the oscillations become stronger, the χ*^J^* parameter or the integral may rise again, after a decrease in first iterations.

#### Implementation of the unfolding procedure

Because the spectra of novel scintillation detectors (with a crystal made of CeBr_3_ for example) have an energy resolution of a few percent, a spectrum resolution of 4096 channels was chosen. This was decided as the energy scale of the spectrum bins has to be linear, and an energy range up to 10 MeV has to be covered with a sufficient resolution at low energies (<100 keV), at the same time. GRAVEL is included in PTB’s UMG package, which is an open-source code written in Fortran. To overcome the limitations of the UMG code, the algorithm was included in the SpecConvert software. This Visual Basic code also includes features which are needed in this context. Such features comprise the quasi-continuous shrinking or stretching of both axes of a spectrum, the summing of spectra (including the automatic adaption in the case of an energy drift), shifting the energy axis, multi-parameter fitting of several added spectra to a reference spectrum, matrix inversion, the calculation of splines and more. Several response matrices for different detector configurations (defined above) were calculated by using MCNP 6.2. The simulated spectra (representing columns of the response matrix) are folded with the detector resolution. The folding has the effect of a spline that makes the ripple of the spectra caused by statistical uncertainties smooth.

Difference spectra of foreground and background measurements may include a number of channels with negative counts. To remove negative counts in an input spectrum, the negative values may just be ignored. Alternatively, an algorithm can be used which shifts negative counts to higher channels until positive numbers remain. This is done by adding the negative counts of one channel to the counts of the next higher channel, until at least zero content is reached. This algorithm compensates, for example, perfectly for statistical oscillations around the zero level. In such a case, a clear positive artefact would remain if only the negative counts were ignored. Alternatively, also a spline of the input spectrum can be unfolded. A spline will automatically remove most zero channel entries, so that the needed corrections will be smaller. Finally, the unfolded spectra have to be normalised to obtain fluence spectra in defined units, such as photons per cm^2^ at a certain distance from the target. In order to do this, the actual entrance area of the detector crystal and its distance from the target have to be taken into account. This translation cannot be included in the response matrix. Optionally, the fluence spectra are converted into dose spectra by applying the relevant fluence to dose conversion coefficients as a last step.

#### Examples of unfolded spectra

**Figure 12:**
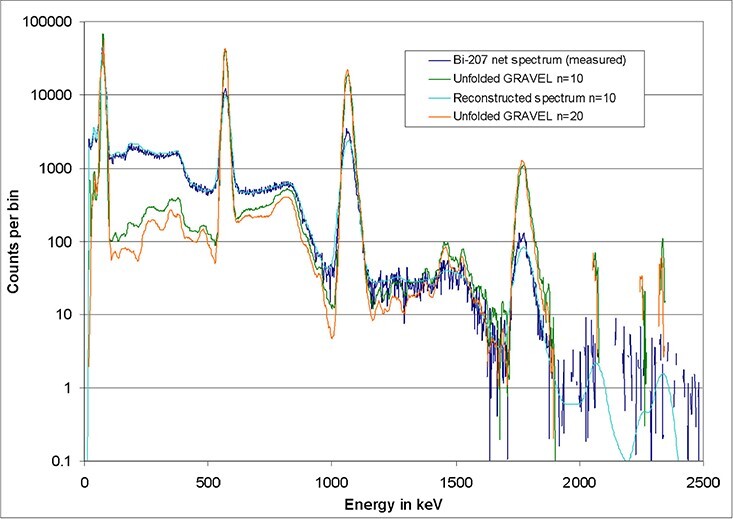
Unfolding of a measured spectrum based on a spline of the measured spectrum as a first estimate.

The following figures illustrate typical results of unfolded spectra and reconstructed spectra (‘test spectra’). A CeBr_3_ detector with a crystal of the size 1.5″ × 1.5″ was irradiated with a ^207^Bi source. A background measurement (performed without source) has been subtracted. Neglecting uncertainties, this net spectrum is purely the result of the ^207^Bi emission lines at 80 keV (three unresolved X-ray lines), 570, 1064 and 1770 keV. If a spline of the measured spectrum serves as a first estimate of the GRAVEL algorithm, results like those shown in Figure 12 are obtained. Already after 10 deconvolution loops, a good result is reached.

The curve in light blue, the test spectrum (i.e. the unfolded spectrum times the response matrix), is adapted well to the measured curve by the algorithm. According to the GRAVEL formula, structures with high count rates are modified preferably, while the background is more or less defined by the first estimate. Channels with high count numbers, especially in peak regions, are modified by unfolding, so that they are clearly elevated on the background. On the contrary, channels with count numbers <1% of the high count numbers are not lowered considerably. This even applies if the patterns of Compton scattered photons (in short: Compton continua) should be removed by unfolding, because this characteristic of the detector is completely included in the response matrix. The uncertainties in the region of the Compton edge of the 1770 peak produce spurious peaks at 1460 and 1530 keV, which have no physical background. This illustrates that the channels with count numbers <1% of the high count numbers are not necessarily unfolded completely due to the construction of the GRAVEL formulas.

**Figure 13:**
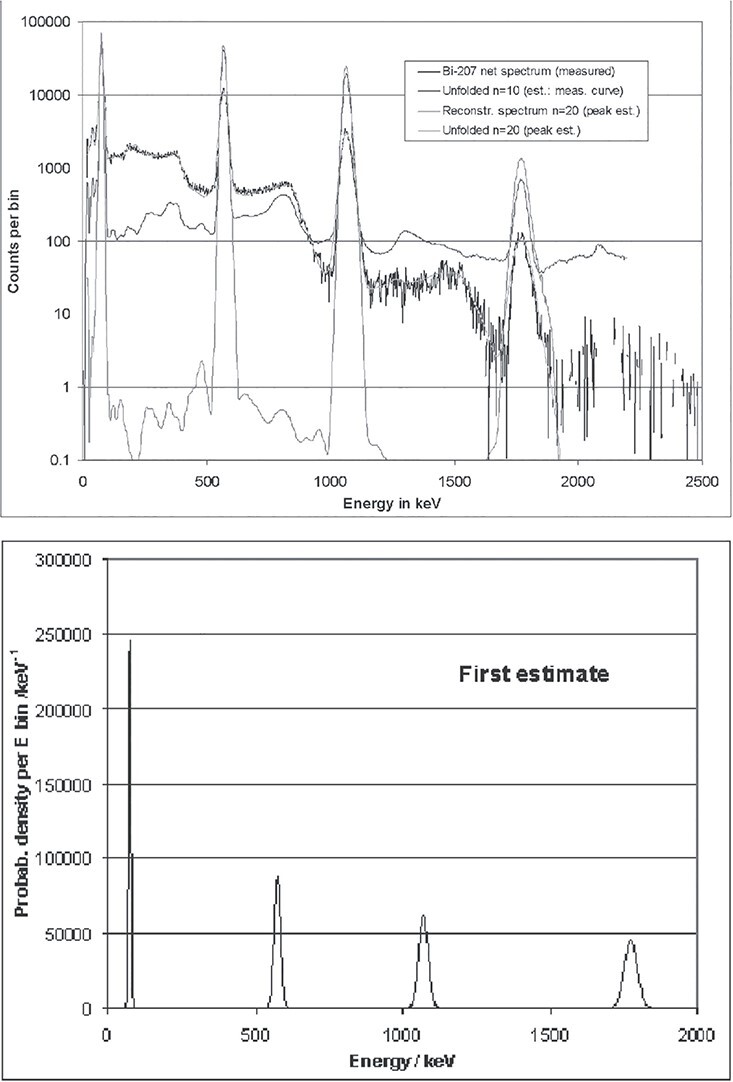
Unfolding of a measured spectrum based on a peak prediction as a first estimate.

Figure 13 shows the result of a different approach. Such a result is obtained when the physical position of the peaks is included in a first estimate (all background channels have the contents 1, not visible in the figure). When doing so, the algorithm does not converge so quickly (the green curve, obtained after 10 iterations, still shows an unphysical peak-to-background ratio). However, after 20 cycles (orange curve), the structures of Compton scattered photons are completely removed, so that almost only the photo peaks remain. Finally, the low background of the first estimate becomes visible. This behaviour is only expected if the first estimate and the measured spectrum are consistent, i.e. have the photo peaks at the same positions. The first estimate has to be constructed with care.

It is important to note that linear plots of the results of both approaches look very similar. The clearly visible differences in the remaining background in Figures 12 and 13 have little influence on the total fluence or the total dose derived from the spectra. The number of iterations as well as the choice of the first estimate only cause an uncertainty of total fluences or doses in the order of a few percent, depending on the concrete case. Relatively higher uncertainties are expected if the photo peaks grow smaller than the escape peaks (here at energies of several MeV). Absolute numbers concerning this effect will be presented in the next section.

## Results

First, the conversion and the unfolding method will be verified separately by using independent reference data. Then, the results of both methods will be compared using relevant example spectra.

### Verification of the spectrum conversion method

The total fluence, air kerma and ambient dose equivalent derived from measured spectra are compared with that derived from the known activities. Either one radioactive source of the isotopes ^137^Cs, ^22^Na and ^207^Bi was installed at a distance of 39 cm from the front window of a CeBr_3_ detector. The experimental installation, which preferably avoids scattering outside of the source and the detector, is identical with the modelled configuration (described earlier). Detectors with both crystal sizes (1″ × 1″ and 1.5″ × 1.5″) are irradiated, so that six data sets are obtained (Table 1).

**Table 1:** The evaluation of six recorded spectra (using two different detectors and three radioactive sources).

			Activity-based calculation (reference)			Conversion of measured spectra		
No.	Nuclide	Crystal size	Tot. Φ	Tot *K_a_*	Tot H^*^(10)	Tot Φ (E > 30 keV)	Tot *K_a_*	Tot H^*^(10)
			cm^−2^	nGy	nSv	cm^−2^	nGy	nSv
1	Cs-137	1.0″ × 1.0″	22 372	65.3	78.5	23 480	65.1	78.8
2	Na-22	1.0″ × 1.0″	5575	19.5	23.2	6728	21.5	25.4
3	Bi-207	1.0″ × 1.0″	60 635	163.7	196.9	64 473	167.6	200.6
4	Cs-137	1.5″ × 1.5”	33 461	97.7	117.4	33 749	95.5	113.6
5	Na-22	1.5″ × 1.5”	7188	25.2	30.0	6870	23.6	28.0
6	Bi-207	1.5″ × 1.5″	90 701	244.9	294.6	94 794	247.8	296.5

Reference values (derived from activities) and data of evaluated spectra using the conversion method are listed.

For the calculation of total fluences or doses, the contents of each channel of a measured spectrum is multiplied by the energy and by the conversion coefficient *w_i_* of this channel. In addition, the total fluence, air kerma and H^*^(10) are calculated. Spectrum conversion does not lead to an unfolded spectrum. The conversion method produces spectra whose integral is identical with a measurand, like the total fluence or the total H^*^(10), depending on the derivation of the conversion coefficients. The shape of the fluence, air kerma and H^*^(10) curves, however, has no deeper meaning.

For the purpose of verification, the expected fluence, air kerma and ambient dose equivalent were calculated on the basis of the known distance between the detector and the source, the irradiation time, the detector crystal size and the activity of the sources. The assumption of an effective, energy-independent position of the detector may lead to an adjustment of the calibration factor derived in the following. In Table 1, all calculated fluence and dose values are listed in comparison so that the data derived by using the conversion method are verified. The absolute numbers listed in Table 1 are related to the concrete experiment (activities, measuring times and geometry) and are listed only for the sake of a relative comparison below. Assuming that the data based on the source activities are correct, calibration factors are derived for the detector system with dosimetric read-out by spectrum conversion. The calibration factors are defined as the mean of the measured fluence, kerma and H^*^(10), in each case divided by the calculated corresponding reference value. Table 2 shows the obtained calibration factors and the difference of the fluence and dose values listed in Table 1 to ease the comparison.

**Table 2:** Derived calibration factors and the ratio of calculated reference values and converted, measured spectra.

			Mean		Ratio calculated/measured		
No.	Nuclide	Crystal size	*k*_cal_	u(*k*_cal_)	Fluence	Air kerma	*H* ^*^(10)
1	Cs-137	1.0″ × 1.0″	0.98	5.9%	0.95	1.00	1.00
2	Na-22	1.0″ × 1.0″	0.88	11.0%	0.83	0.91	0.91
3	Bi-207	1.0″×1.0″	0.97	5.1%	0.94	0.98	0.98
4	Cs-137	1.5″ × 1.5″	1.02	4.7%	0.99	1.02	1.03
5	Na-22	1.5″ × 1.5″	1.06	3.1%	1.05	1.07	1.07
6	Bi-207	1.5″ × 1.5″	0.98	4.5%	0.96	0.99	0.99

The fluence and dose values derived from ^137^Cs and ^207^Bi spectra by using the conversion method differ by <5% from the reference values. Higher deviations are found when the ^22^Na data are compared. The reason for this is that this source emits positrons, which subsequently generate 511 keV photon pairs by annihilation, partly already inside the source, partly in the surroundings of the detector. The emission probability of 511 keV photons directly from the sources is not exactly known. It will partly depend on the construction of the source. Furthermore, the pair production outside of the source is not included in the calculation of the reference values (the latter are only valid for the photon content). Therefore, the reference values concerning the ^22^Na source are not well known. As a conclusion, ^22^Na sources are not useful as calibration sources if the events of the 511 keV peak enter the result of an evaluation, which is the case here.

### Verification of the GRAVEL unfolding method

Analogous to the verification of the conversion method, the same ^137^Cs, ^22^Na and ^207^Bi spectra are unfolded to calculate the total fluence, air kerma and ambient dose equivalent. The same spectra are now unfolded and evaluated by using the GRAVEL algorithm implemented in SpecConvert.

First, the energy scale of the measured spectra has to be adapted to the energy scale of the detector response matrix used (this step is not necessary if the conversion method is used). A region of interest, which has to be unfolded, has to be chosen (this covers all relevant photo peaks and their corresponding Compton continua down to 20 keV). To gain information on each iterative step, SpecConvert writes a protocol including the results of each iteration step, inclusive of the parameter χ and the integrated counts of the spectrum. For the ease of evaluation, the changes in both parameters, representing the first derivative, are additionally observed. In many cases, both parameters do not undergo a minimum, but approach a limit value. However, beyond a certain iteration step, normally found around 10, the unfolded curve starts to oscillate visibly, which is unphysical. The first iteration already approaches the final unfolded curve very closely if a realistic first estimate is chosen as input. Therefore, the decision on the reasonable final iterative step, which leads to the best unfolded curve, is uncritical. Quantitative investigations of this statement and other observations of this work is published in a dedicated paper on unfolding^(17)^.

**Figure 14:**
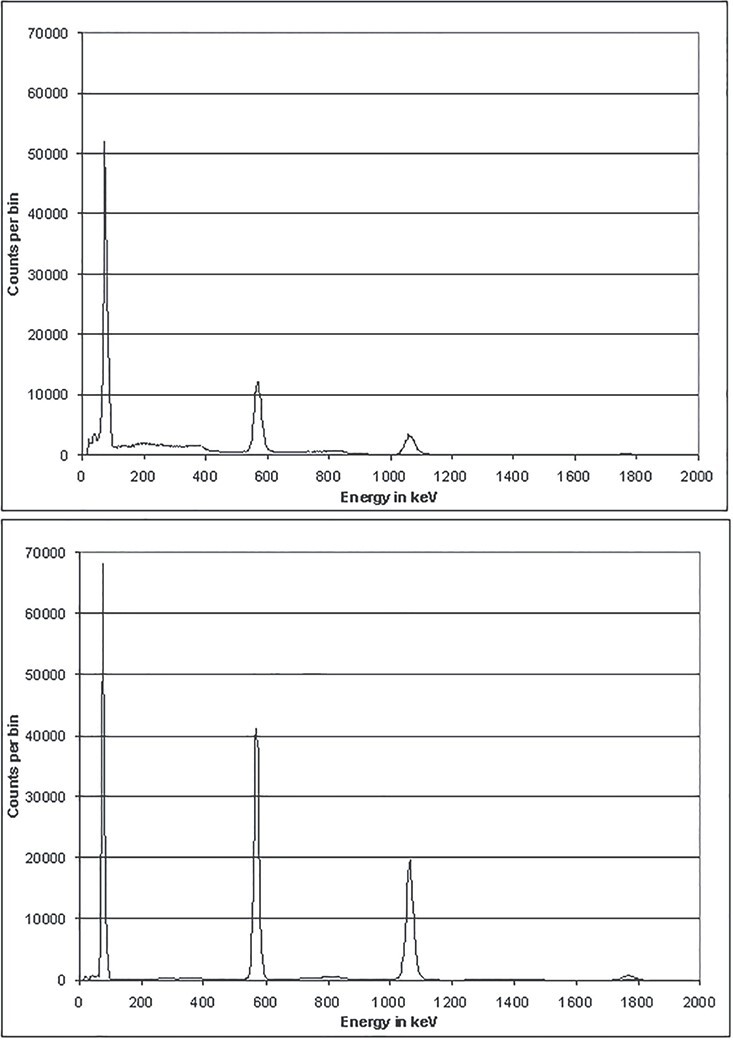
Example of a measured spectrum of a CeBr_3_ detector (with a 1.5″ × 1.5″ crystal) irradiated with a ^207^Bi source (top) and the corresponding unfolded spectrum (bottom).

The result of the unfolding of a measured ^207^Bi spectrum is illustrated in Figure 14. The consequence of unfolding is that the Compton continua are removed from the spectrum, thus showing the natural emission lines of the ^207^Bi isotope. In this way, the fluence at the position of the detector is obtained. The fluence spectrum can be converted into dose quantities by using fluence-to-dose conversion coefficients. Together with the reference data (see the section above) the results are listed in Tables 3 and 4 for the purpose of comparison.

**Table 3:** The evaluation of six recorded spectra (using two different detectors and three radioactive sources)

			Activity-based calculation (reference)			Unfolding of measured spectra		
No.	Nuclide	Crystal size	Tot. Φ	Tot *K_a_*	Tot H^*^(10)	Tot Φ ^#)^	Tot *K_a_*	Tot H^*^(10)
			cm^−2^	nGy	nSv	cm^−2^	nGy	nSv
1	Cs-137	1.0″ × 1.0″	22 372	65.3	78.5	22 354	60.4	72.7
2	Na-22	1.0″ × 1.0″	5575	19.5	23.2	6192	19.5	23.3
3	Bi-207	1.0″ × 1.0″	60 635	163.7	196.9	59 620	148.7	179.9
4	Cs-137	1.5″ × 1.5″	33 461	97.7	117.4	31 629	88.8	106.9
5	Na-22	1.5″ × 1.5″	7188	25.2	30.0	6636	23.3	27.7
6	Bi-207	1.5″ × 1.5″	90 701	244.9	294.6	87 965	227.8	274.8

Reference values (derived from activities) and data of evaluated spectra using unfolding are listed. #) means energy range > 30 keV.

**Table 4:** Derived calibration factors and the ratio of calculated reference values and unfolded spectra.

			Mean		Ratio calculated/measured		
No.	Nuclide	Crystal size	*k*_cal_	u(*k*_cal_)	Fluence	Air kerma	*H* ^*^(10)
1	Cs-137	1.0″ × 1.0″	1.05	9.0%	1.00	1.08	1.08
2	Na-22	1.0″ × 1.0″	0.97	12.0%	0.90	1.00	1.00
3	Bi-207	1.0″ × 1.0″	1.07	8.9%	1.02	1.10	1.09
4	Cs-137	1.5″ × 1.5″	1.09	4.8%	1.06	1.10	1.10
5	Na-22	1.5″ × 1.5″	1.08	2.0%	1.08	1.08	1.08
6	Bi-207	1.5″ × 1.5″	1.06	5.0%	1.03	1.07	1.07

The obtained results and figures of the unfolded spectra look reasonable. The removal of Compton continua, which are almost completely produced by the detector, is expected. The results tabulated in Tables 3 and 4 deviate from the reference values by 10%, at maximum. The calibration factor *k*_cal_ of both detectors, the quotient of the reference air kerma (calculated from the activity) divided by the measured air kerma, is close to 1.08 as a systematic effect, when doses are calculated. The data of the measurements of the ^22^Na spectrum are excluded because the reference values of this source are uncertain (as explained in detail in the last section). A similar systematic difference between the data of both detectors was also discovered when the conversion method was applied. The reason may be a slight deviation of the actual crystal sizes from the drawings. Second, edge effects exist because of the divergence of the photon rays. When photons are emitted from a point source, a cone-shaped beam hits the detector front. Photons are detected with a smaller efficiency near the edge because less material is available in this direction (going to zero exactly at the edge). Such effects are more relevant for a small detector crystal because the entrance window of a small crystal has a relatively longer edge (the surface/volume ratio of a small crystal is less favourable).

Furthermore, the position of the effective centre of the crystal (which is needed to calculate fluences and doses at the position of the crystal), affects the calibration factor directly. Physically, the effective detection position lies inside the crystal and depends on the photon energy. However, for simplification, one fixed position is defined instead (1 cm behind the front of the crystal), so that one spectrum can be recorded at one defined location. All three described effects in combination have an influence on the calibration factor, which is derived empirically here by the calculation of the ratio of reference data and processed measured data. The data in Tables 3 and 4 may serve as an experimental verification of the correct implementation and use of the GRAVEL algorithm. Further evidence is found when both the results of the conversion method and the unfolding method are compared in the investigations below.

### Comparison of the results of both methods

For verification purposes on the basis of examples, the data from the investigations of the spectra of radioactive sources are compared first of all. Table 5 illustrates that the conversion method leads to systematically higher values in this case. For both detectors, the mean difference is ~8% if the ^22^Na data are not taken into account because of their higher uncertainty. One reason for this is that the background by electrons and positrons, which is emitted in addition to the photon radiation, is also transferred to fluence or dose by the conversion method, while the response matrix used for the unfolding is only calculated for pure photon radiation. Total fluence or dose data derived from unfolding have an uncertainty of ~5% and data derived from conversion of ~3% ^(17)^. The uncertainty budgets include systematic uncertainties (of the type B). Especially the results obtained by unfolding depend on the difference of the assumed and actual detector resolution of a measurement. Because of these reasons, a systematic difference of 8%, as found here, is in agreement with a correct application of both methods.

**Table 5:** Ratios of values obtained by conversion and unfolding (the values themselves are listed in Table 1 and Table 3).

			Ratio conversion/unfolding		
No.	Nuclide	Crystal size	Fluence	Air kerma	*H* ^*^(10)
1	Cs-137	1.0″ × 1.0″	1.05	1.08	1.08
2	Na-22	1.0″ × 1.0″	1.09	1.10	1.09
3	Bi-207	1.0″ × 1.0″	1.08	1.13	1.11
4	Cs-137	1.5″ × 1.5″	1.07	1.08	1.06
5	Na-22	1.5″ × 1.5″	1.04	1.01	1.01
6	Bi-207	1.5″ × 1.5″	1.08	1.09	1.08

The results of the conversion method and the unfolding method agree well within the uncertainties of both methods. The conversion method is more flexible because its results are less model-dependent. In addition, the uncertainty caused by the necessity to select the first estimate is a disadvantage of the unfolding method. On the other hand, the shape of a fluence spectrum (without any influence of the detector) can only be obtained by unfolding. A more detailed study on uncertainties is published separately^(17)^.

### Evaluation of example photon spectra recorded at PTB’s ion accelerator facility

In the following, two examples of the measurement of spectra at the PTB Tandetron accelerator are discussed. A scintillation detector based on a 1.5″ × 1.5″ CeBr_3_ crystal is used for the recording of spectra.

#### Unfolding of measured spectra in the R-F photon field

**Figure 15:**
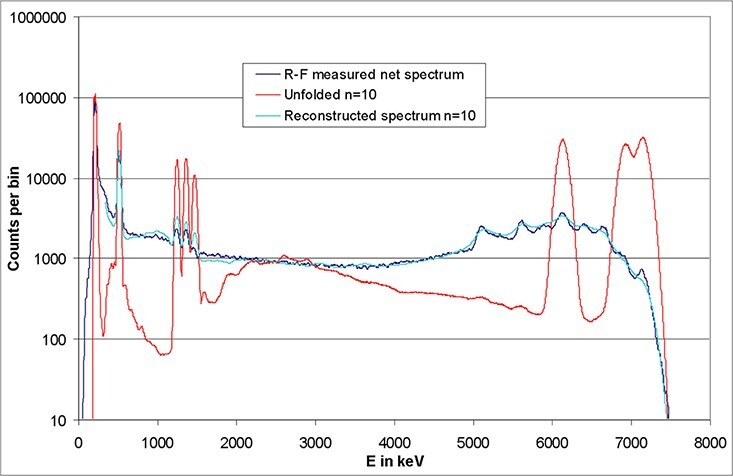
Measured R-F photon spectrum by using a CeBr_3_ detector (crystal size: 1.5″ × 1.5″) in dark blue. The result of the unfolding (after 10 iterations) is displayed in red. The reconstructed test spectrum is plotted in light blue. The unfolded spectrum has to be re-normalised to obtain a fluence spectrum.

At PTB, the ISO R-F quality is realized by using a linear Tandetron accelerator to bombard a CaF target with protons having energy of ~2.7 MeV. The target emits three photon lines at 6.1, 6.9 and 7.1 MeV (Figure 15), so that a high-energy photon field is produced with a mean energy of ~6.6 MeV referring to H^*^(10). When the high-energy photons hit the detector, one or two 511 keV photons can escape without being detected. Thus, six escape lines are produced, in addition. Therefore, nine lines are expected, in total, of which eight lines are observed in the measured spectrum because two of the lines have nearly the same energy. Other ^19^F emission lines are observed at 197, 1235 and 1355 keV (two unresolved lines) and 1459 keV (the line expected at 110 keV is not visible in Figure 15). Furthermore, a 511 keV positron annihilation line is observed as the consequence of pair production by the high-energy photons and the decay of an excited ^16^O state in the target into electron/positron pairs. The electrons and positrons have an energy up to 4.5 MeV, with a distribution which peaks at ~3 MeV. Here and in all other spectra, the background which was obtained by performing a shadow cone measurement was subtracted.

**Figure 16:**
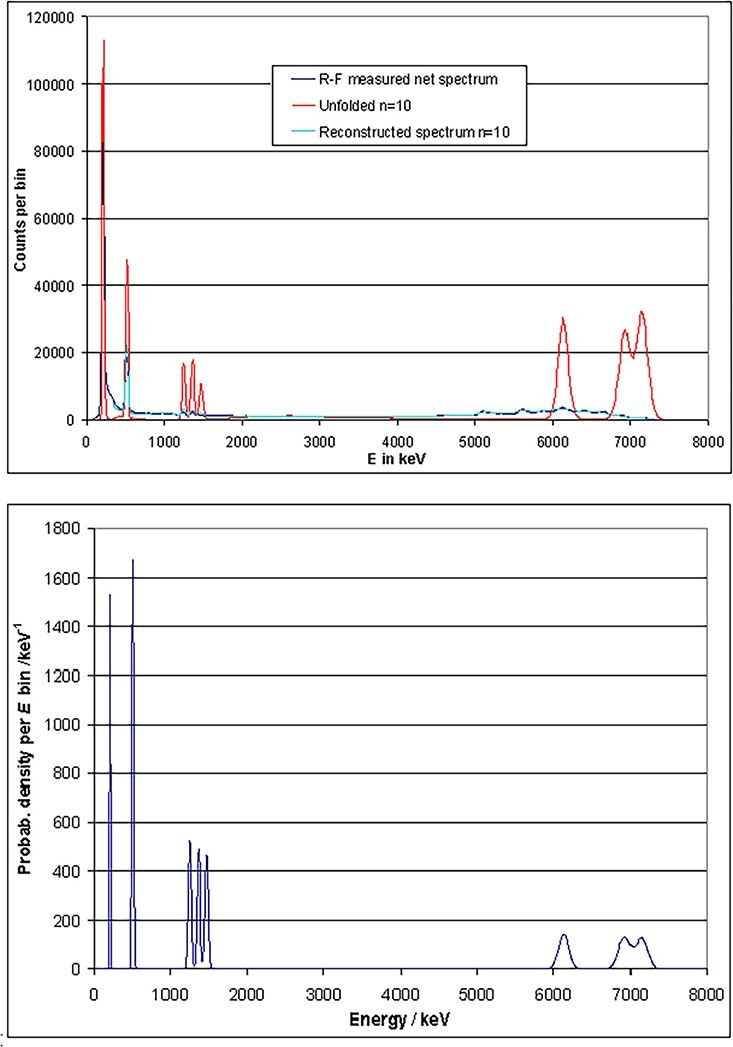
Linear plot of Figure 15 (top) and of the first estimate of the unfolding procedure (bottom).

The known gamma lines are used as a first estimate for unfolding (Figure 16, top). The background level is set to 1, so that unfolding in all channels is possible (according to the GRAVEL formulas, only the ratio peak to background is relevant). The area below all peaks is identical, but the peak heights look different because the detector resolution increases with energy. The resulting spectrum after unfolding is also shown in Figure 15, together with the measured spectrum. Iteration 10 yields the smallest χ, so that this result is regarded as the best estimate of the undisturbed fluence spectrum at the position of the detector (further iterations will produce unphysical oscillations, which result in an increasing χ).

After unfolding, only the three actual emission lines remain at high energies. The Compton continua are drastically lowered in the whole spectrum. Between 2 and 4 MeV, there seems to be a very wide peak. This coincides with the spectrum expected from pair production in the target (the energy distribution of the electrons is detected). The linear plot (Figure 16, bottom) illustrates the removal of the continuous background through unfolding more clearly. Structures at a level of <1% of the peaks heights are, however, questionable, as pointed out above. Hence, a quantitative evaluation at such a low level is not possible. To summarise, the results described in this section demonstrate the capability and limits of the GRAVEL unfolding method through examples.

#### Evaluation of photon spectra recorded in the ISO 144 keV neutron field

**Figure 17:**
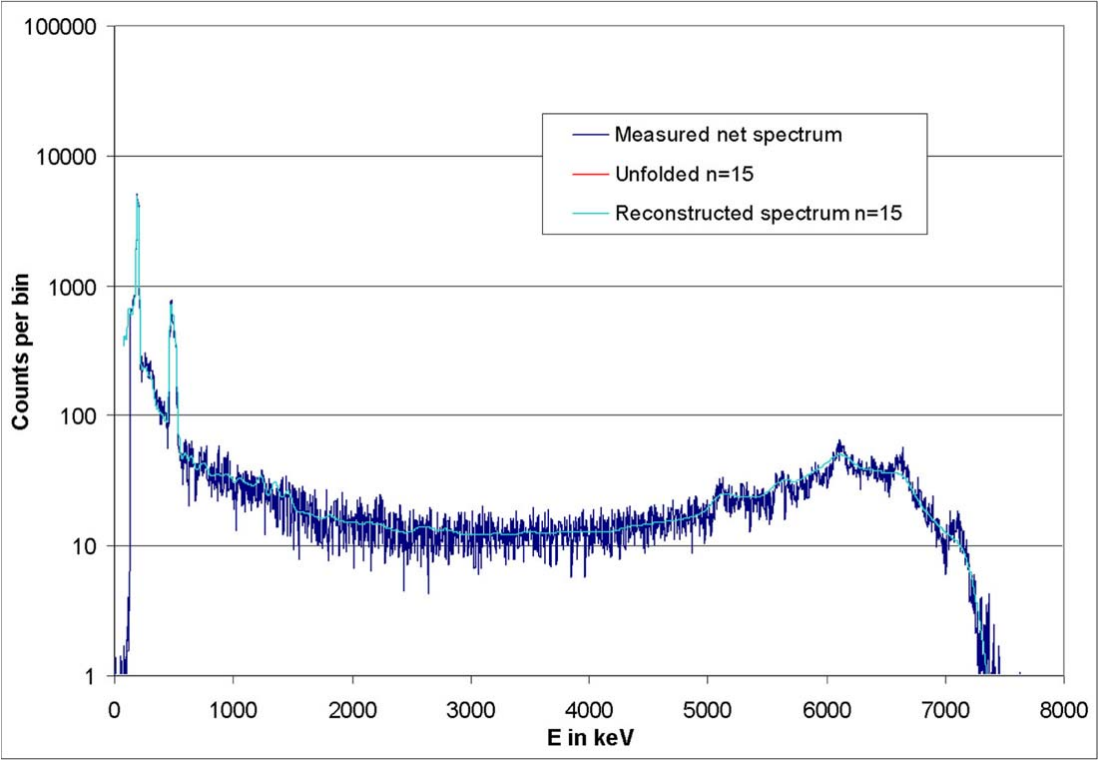
Photon spectrum measured in 144 keV ISO neutron field, recorded by using a CeBr_3_ detector (crystal size: 1.5″ × 1.5″) in dark blue. The result of the unfolding (after 15 iterations) is displayed in red. The reconstructed test spectrum is plotted in light blue.

Generally, 144 keV neutrons are either produced by bombarding a LiF or a Li target with protons. Because a Li target is difficult to handle due to its high chemical reactivity, often a stable LiF target is used. The disadvantage of the LiF target is that the fluorine emits a significant amount of high-energy photons, so that the photon contamination of the neutron field is not negligible. This is illustrated in Figure 17 (dark blue curve), where a net spectrum is shown which is obtained from a measured foreground spectrum after subtracting a background spectrum recorded behind a shadow cone. The photon spectrum was separated from the neutron-induced detector output by applying the time-of-flight method. A presupposition of the method is a pulsed accelerator beam, which produces proton bunches of a few ns, hitting the target in a defined frequency (here: 125 MHz). The events induced by direct photons and neutrons are separated by the different flight time of the photons and neutrons after a proton bunch hits the target.

**Figure 18:**
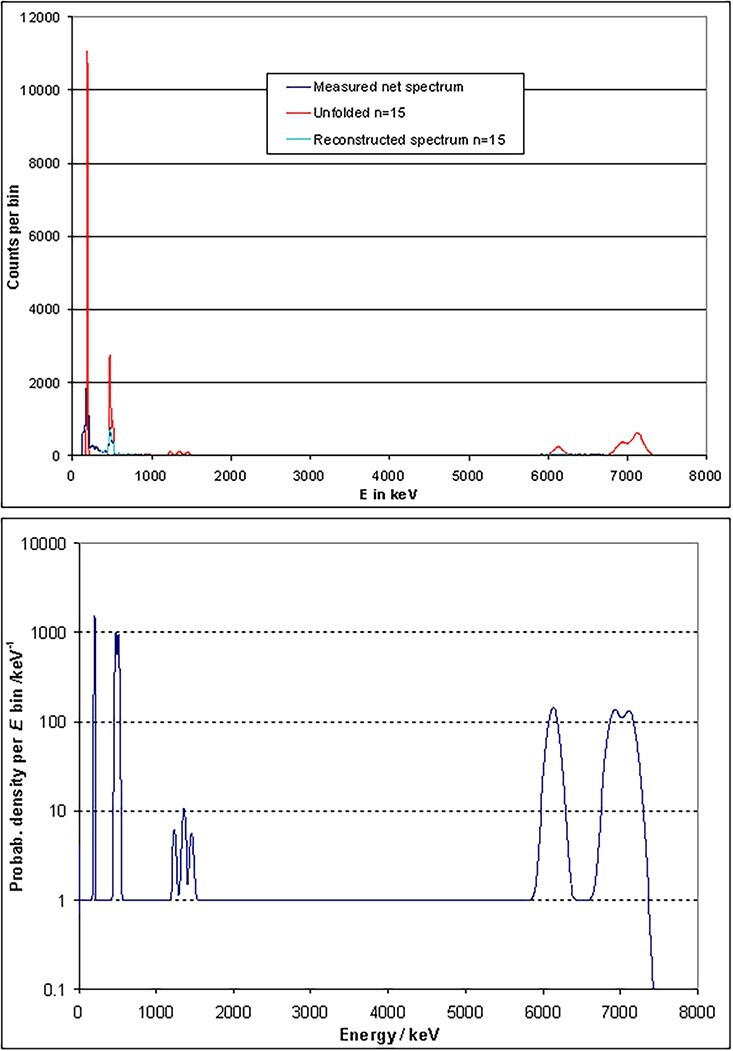
Linear plot of Figure 17 (top) and of the first estimate of the unfolding procedure (bottom).

In addition to the application of the conversion method to obtain the total fluence and dose, unfolding by GRAVEL was also performed. The first estimate (Figure 18, bottom) leads to the unfolded curve in Figure 17 (in red) after 15 iterations. In addition, this figure shows the reconstructed spectrum (in light blue), which agrees quite well with the measured spectrum (in dark blue). When these spectra are plotted on a linear scale (Figure 18, top), the emphasis of the unfolding on the photo peaks is more evident. Structures produced by the detector, especially the Compton continua and escape peaks, are removed by unfolding. From the unfolded spectrum, the total fluence, air kerma and H^*^(10) are calculated by applying the fluence to dose conversion curves (Figures 20–22). After applying both methods, conversion and unfolding, to three different ISO fields, the resulting data are listed in Table 6. They agree very well within a range of ±4%, which can be seen in Table 7. There is no clear tendency visible that either method leads to higher or lower resulting values.

**Figure 19:**
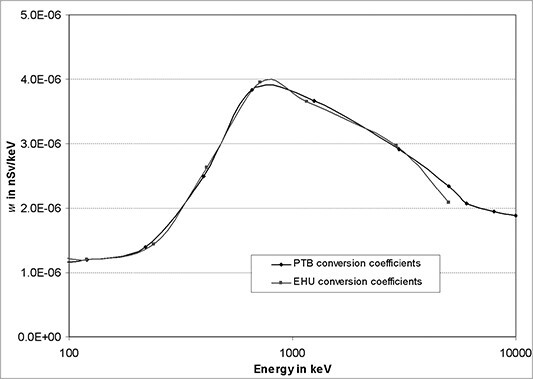
*H* ^*^(10) conversion coefficients valid for a CeBr_3_ detector with a 1″ × 1″ crystal calculated independently by two institutions, PTB and EHU^(18)^.

**Figure 20:**
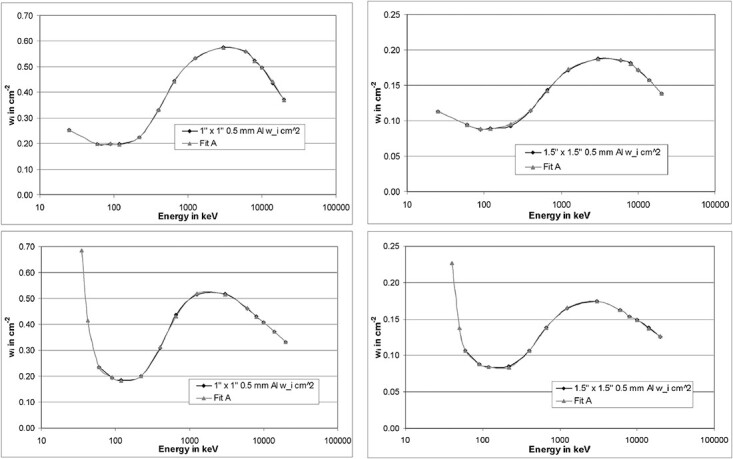
Left curves: spectrum to fluence conversion curves for a detector with a 1″ × 1″ crystal combined with a 0.5 mm Al casing (top) and a 5 mm Al casing (bottom). Right curves: likewise, but for a detector with a 1.5″ × 1.5″ crystal.

**Figure 21:**
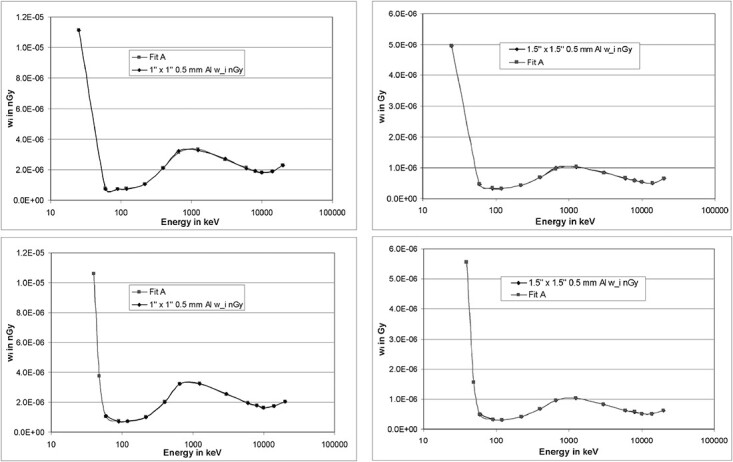
Left curves: spectrum to air kerma conversion curves for a detector with a 1″ × 1″ crystal combined with a 0.5 mm Al casing (top) and a 5 mm Al casing (bottom). Right curves: likewise, but for a detector with a 1.5″ × 1.5″ crystal.

**Figure 22:**
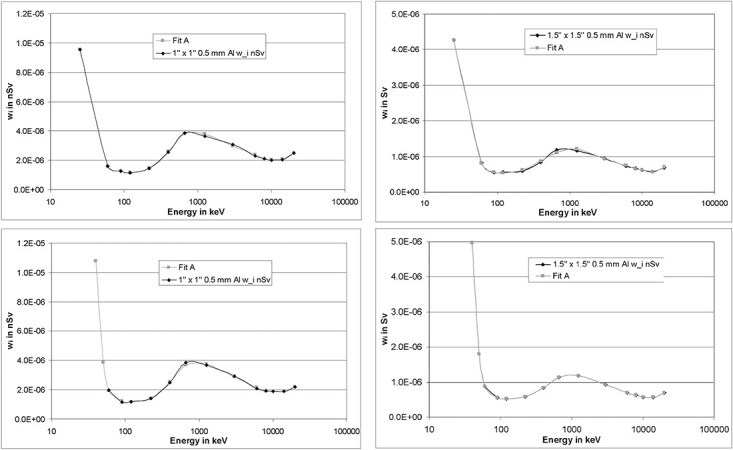
Left curves: spectrum to H^*^(10) conversion curves for a detector with a 1″ × 1″ crystal combined with a 0.5 mm Al casing (top) and a 5 mm Al casing (bottom). Right curves: likewise, but for a detector with a 1.5″ × 1.5″ crystal.

**Table 6:** The evaluation of three photon spectra, which were recorded in a 144 keV neutron field at three angles, resulting in three different neutron energies.

			Unfolding of measured spectra		
No.	Energy	Angle	Tot. Φ	Tot. K_a_	Tot H^*^(10)
	keV	deg	cm^-2^	nGy	nSv
1	24	76.5	17.6	0.70	0.78
2	71	50	19.6	0.73	0.83
3	144	0	22.6	0.77	0.87
			Conversion of measured spectra		
No.	Energy	Angle	Tot. Φ	Tot. K_a_	Tot H* ^(10)^
	keV	deg	cm-2	nGy	nSv
1	24	76.5	16.9	0.68	0.78
2	71	50	20.2	0.72	0.81
3	144	0	21.7	0.77	0.88

Data of evaluated spectra using the GRAVEL unfolding method and the conversion method are listed.

**Table 7:** The ratio of the integral of the unfolded and of the converted measured spectra.

			Ratio unfolded/converted spectra		
No.	Energy	Angle	Fluence	Air kerma	H* (10)
	keV	deg			
1	24	76.5	0.96	0.98	1.00
2	71	50	1.03	0.99	0.98
3	144	0	0.96	1.00	1.02

## Discussion of pros and cons of each method

In all cases where the total fluence or dose results of the conversion method were compared with those of the unfolding method, a good agreement was achieved. The uncertainties of each method are only in the order of a few percent. When systematic deviations are found, one reason may be the following effect. The calculated total fluence and dose depend on the considered energy range, which is covered by the integration. The conversion method is based on the assumption that any photo peak is recorded in combination with the associated region of Compton scattered photons. But when a photo peak lies slightly above the lower threshold of the integral, the associated energy region of Compton scattered photons is, however, cut off. Thus, the integrated result will be low. Vice versa, unfolding will also elevate every clear photo peak, even if one is found slightly above the lower energy threshold. Hence, this method may lead to comparably high values, because it indirectly extends the energy range even down to energies where no measured data exist. However, this effect is only observed if the Compton cross-section is considerable in comparison with the photo cross-section, in this case (using a CeBr_3_ detector) above 150 keV.

In comparison with unfolding, the conversion method is less model-dependent. If there are structures in the spectrum which are not caused by photons (non-regular case), the referring events will be converted independently of their origin, though the physical model and, hence, the referring conversion coefficient, are not completely correct. However, the detection of electrons is also partly incorporated in a model which includes, first of all, only the emission of photons from a source. This is because a secondary electron equilibrium will be established inside the detector at the latest. One has to keep in mind that the photons only interact with the scintillation crystal indirectly, via electrons. Therefore, pure electron radiation will lose some energy on its way through the detector casing into the scintillation crystal, but in the scenarios described above, only a small reduction of the measured dose is expected because the casing of the detector used is thin, i.e. 0.5 mm.

Before applying the conversion method, a calculation of the net spectrum (by subtracting the back-ground) is not necessary. The foreground and the background spectrum can be converted to fluences and doses separately, and afterwards the difference of the integrated spectra can be calculated. This approach avoids the calculation of difference spectra with negative count values because of statistical reasons. Nevertheless, the conversion method will even handle negative entries in bins correctly. Unfolding, on the contrary, is neither possible if elements of the response matrix are negative (which should not occur), nor if spectrum entries are negative or zero. Therefore, negative spectrum bins have to be removed before unfolding. This will cause an additional uncertainty. The procedure proposed above minimises this problem by (a) unfolding a spline of the measured spectrum, which reduces the number of negative counts and (b) pushing the negative count values up to higher positive values, so that a statistical oscillation around the zero level is flattened or even removed. Both mechanisms can be used optionally when the SpecConvert code is used. Especially mechanism (b) is preferable, rather than just cutting away negative entries, because the latter will generate a false positive signal in the case that the spectrum just oscillates around a level near or at zero.

The application of the unfolding method requires much higher efforts, because the calculation of a response matrix with a high resolution consumes extensive computer power and takes time (depending on the resources and the size of the matrix). A further disadvantage is that the energy scale of each measured spectrum has to be adapted to the energy scale of the response matrix. Therefore, a reliable algorithm to shrink or stretch spectra quasi-continuously is needed (two methods are implemented in SpecConvert). However, a correct energy calibration is an important prerequisite of both discussed approaches.

Unfolding by using the GRAVEL algorithm can reproduce the undisturbed fluence spectrum at the position of the detector. In all cases where such a ‘natural’ spectrum is needed, unfolding is inevitable. The results are especially useful if the measured spectrum already shows photo peaks (of a sufficient resolution). Even the peak areas, i.e. the undisturbed spectral fluence distributions at the detector position, can be reconstructed from such spectra if either the irradiation geometry is known or the response of the detector is isotropic. Uncertainties may be considerable if pronounced escape peaks are present. Overlapping or coinciding peaks may especially create ambiguities concerning the evolution of single peaks. Especially in such a case, the results may be biased by the first estimate of the deconvolution. *A priori* knowledge of expected emission lines will lessen this problem.

A high precision of derived fluence and dose values is expected if the method of spectrum conversion is chosen together with a detector-specific calibration factor, which was determined experimentally. The calibration factor will cover systematic deviations of measured results which are caused by a number of uncertainties, such as the limited knowledge of the geometrical model and the exact composition of the detector materials.

## Conclusion and summary

Both the conversion method and the GRAVEL unfolding method are powerful techniques to calculate fluences and doses from spectra. Especially when both methods are combined, the consistency of the results can be checked as an indicator. This may disclose errors in the complex data evaluation process. The application of both methods was shown by means of examples, which are all based on measured spectra of CeBr_3_ detectors. This type of scintillation detector has an energy resolution far superior to that of a conventional NaI detector. Therefore, it rather allows the detection of photo peaks, which is useful if unknown spectra are to be analysed. For the implementation of both methods, MC simulations are essential because the detector response to mono-energetic photons has to be known in a wide energy range. In this work, the MCNP code was used to calculate absolute responses, after verifying the correctness of the implementation of the code. Measured spectra could be simulated with a very high agreement.

The application of the conversion method and/or unfolding allow the use of a spectrometer as a spectro-dosemeter. The replacement of a conventional dosemeter by a spectrometer will lead to a considerable increase in information. In this work, despite the simplifications of both approaches, the uncertainty of calculated fluences or doses is <5% or even better, so that a higher precision than that of a conventional dosemeter is reached in a wide energy range (20 keV to 20 MeV). Furthermore, traditional dosemeters like ionisation chambers or proportional counters can merely display results in terms of one quantity, for which they were mechanically built.

An important goal, for which the methods of spectrum conversion and unfolding were implemented in this work, is the investigation of photon spectra in standard ISO neutron fields in a future work. A comparison of the results of both methods will help to exclude errors and will serve as verification by consistency. Disagreements will indirectly reveal some information on uncertainties.

As a conclusion, the application of spectro-dosemeters as secondary standards instead of conventional dosemeters is favourable. This is because the evaluation of measured spectra makes it possible to flatten the energy response of the dose (rate) instrument just by using software. Consequently, the uncertainty on the combined angular response and energy response can be pushed down <2% in a wide energy range. This is not accessible by using a conventional dosemeter (a dosemeter which conforms to the standard EN IEC 60846 may show a combined angular and energy response up to 40%). When spectro-dosemeters are characterised correctly, their results can reach a much higher precision in dose (rate) measurements than that of conventional dosemeters.

## Data Availability

All data published and displayed in this article are available and stored permanently at PTB data processing systems.

## A. ANNEX

In the tables below, calculated conversion coefficients *w_i_* are listed, so that three quantities can be calculated from spectra, i.e. the fluence Φ, the air kerma *K*_a,_ the ambient dose equivalent, *H*^*^(10) and the personal dose equivalent *H*_p_ (10). The latter is valid for an angle of incidence of 0°. Fits of these coefficients represent the conversion curves which were used in this work to convert measured spectra to fluence values and doses. The counts of the bins of any spectrum serve as an input for Equation 2.3, assuming that a correct energy calibration exists. A mean dose rate is calculated from a dose value by taking the measuring time of the respective spectrum into account (dose divided by time).

The stated precision of the conversion coefficients listed below is based on the following arguments. The MC simulations, from which peak efficiencies were derived, were performed in Penelope and in MCNP 6.2 as well. The obtained differences are <2% concerning one coefficient for one photon energy. Regarding one example, the good agreement between the simulations is illustrated graphically in Figure 2. When realistic spectra are converted, involving many conversion coefficients, the deviations of single conversion coefficients from ideal values are partly cancelled out because some coefficients are slightly high, others slightly low. Hence, an agreement of >99% is expected. The procedure of the derivation of conversion coefficients, depicted in Figure 9, will entail an additional uncertainty. Therefore, as an example, the conversion coefficients to obtain *H*^*^(10) from the spectrum of a CeBr_3_ detector with a 1″ × 1″ crystal were derived independently in this work and by the University of the Basque Country (EHU)^(18)^. Both attained data sets are based on independent MCNP calculations. Each data set is plotted in Figure 19. In the energy range between 100 keV and 3 MeV, a very good agreement is observed. The EHU value at 5 MeV is low because it does not take the counts in the escape peaks into account. When the fitted conversion coefficients of this work are multiplied with all simulations of the obtained spectra of mono-energetic photons, one after another, the deviation of the total dose calculated by applying the conversion coefficients, on the one hand, and from the known reference dose, on the other hand, is always <2%. The reference dose can be computed analytically from the dose per MC input photon. Because in reality a wide spectrum will be converted instead of a nearly mono-energetic MC spectrum, the total deviation of the converted results from an ideal reference will always be <1%, as far as the fit of the conversion coefficients is concerned. Both effects discussed before will add to an uncertainty <2%. This is the largest and most exact data set of conversion coefficients valid for LaBr_3_, CeBr_3_ and SrI_2_ detectors that has been published in the literature, so far.

**Table 8:** List of data needed for the calculation of the conversion factors *w*_*i*_.

*w_i_*	*E_i_*	Energy range	*μ*_en_/*ρ*	*g*_a_	Air kerma	*H* ^*^(10)
No.	keV	keV	cm^2^/g		nGy	nSv
1	25	20–40	2.73E-01	5.76E-05	1.09E-03	9.41E-04
2	60	40–75	3.10E-02	1.39E-04	2.98E-04	5.18E-04
3	90	75–105	2.34E-02	2.09E-04	3.37E-04	5.67E-04
4	120	105–200	2.34E-02	2.79E-04	4.50E-04	7.08E-04
5	220	200–300	2.75E-02	5.17E-04	9.68E-04	1.33E-03
6	400	300–500	2.96E-02	9.54E-04	1.90E-03	2.39E-03
7	660	500–1050	2.92E-02	1.61E-03	3.10E-03	3.72E-03
8	1250	1050–2000	2.67E-02	3.20E-03	5.37E-03	6.22E-03
9	3000	2000–4000	2.06E-02	8.56E-03	9.99E-03	1.12E-02
10	6000	4000–6900	1.64E-02	1.92E-02	1.61E-02	1.79E-02
11	8000	6900–8900	1.52E-02	2.69E-02	2.00E-02	2.22E-02
12	10 000	8900–11 000	1.45E-02	3.45E-02	2.41E-02	2.66E-02
13	14 000	11 000–15 000	1.37E-02	4.94E-02	3.24E-02	3.56E-02
14	20 000	15 000–20 000	1.31E-02	7.09E-02	4.52E-02	4.96E-02

The air kerma and *H*^*^(10) values in the last columns are related to one photon of the energy *E_i_*.

**Table 9:** Conversion coefficients valid for a detector with a cylindrical CeBr_3_ crystal of the dimensions 1″ × 1″ (diameter × length) and a thickness of the crystal casing of 0.5 mm.

		*w_i_* for conversion of counts to quantity …			
	*E*	Φ	*K*_a_	*H* ^*^(10)	*H*_p_ (10)
No.	keV	cm-2	nGy	nSv	nSv
1	25	0.2539	1.111E-05	9.562E-06	9.839E-06
2	60	0.1977	7.389E-07	1.610E-06	1.751E-06
3	90	0.1982	7.414E-07	1.253E-06	1.437E-06
4	120	0.1984	7.430E-07	1.165E-06	1.241E-06
5	220	0.2250	1.050E-06	1.450E-06	1.528E-06
6	400	0.3295	2.093E-06	2.548E-06	2.752E-06
7	660	0.4457	3.242E-06	3.868E-06	3.674E-06
8	1250	0.5322	3.250E-06	3.650E-06	3.853E-06
9	3000	0.5767	2.713E-06	3.068E-06	2.905E-06
10	6000	0.5599	2.084E-06	2.297E-06	2.315E-06
11	8000	0.5230	1.939E-06	2.111E-06	2.147E-06
12	10 000	0.4967	1.807E-06	2.008E-06	2.042E-06
13	14 000	0.4368	1.870E-06	2.047E-06	1.996E-06
14	20 000	0.3724	2.268E-06	2.479E-06	2.345E-06

**Table 10:** Conversion coefficients valid for a detector with a cylindrical CeBr_3_ crystal of the dimensions 1″ × 1″ (diameter × length) and a thickness of the crystal casing of 5.0 mm.

		*w_i_* for conversion of counts to quantity …			
	*E*	Φ	*K*_a_	*H* ^*^(10)	*H*_p_ (10)
No.	keV	cm^-2^	nGy	nSv	nSv
1	25	1.9907	8.714E-05	7.496E-05	7.713E-05
2	60	0.2346	1.059E-06	1.958E-06	2.131E-06
3	90	0.1926	6.898E-07	1.153E-06	1.334E-06
4	120	0.1858	7.244E-07	1.198E-06	1.283E-06
5	220	0.2000	1.000E-06	1.400E-06	1.536E-06
6	400	0.3093	2.006E-06	2.491E-06	2.368E-06
7	660	0.4366	3.228E-06	3.846E-06	4.222E-06
8	1250	0.5148	3.229E-06	3.665E-06	3.629E-06
9	3000	0.5173	2.564E-06	2.924E-06	2.854E-06
10	6000	0.4613	1.921E-06	2.064E-06	2.123E-06
11	8000	0.4305	1.795E-06	1.944E-06	1.978E-06
12	10 000	0.4085	1.629E-06	1.884E-06	1.804E-06
13	14 000	0.3720	1.735E-06	1.897E-06	1.812E-06
14	20 000	0.3320	2.047E-06	2.189E-06	2.246E-06

**Table 11:** Conversion coefficients valid for a detector with a cylindrical CeBr_3_ crystal of the dimensions 1.5″ × 1.5″ (diameter × length) and a thickness of the crystal casing of 0.5 mm.

		*w_i_* for conversion of counts to quantity …			
	*E*	Φ	*K*_a_	*H* ^*^(10)	*H*_p_ (10)
No.	keV	cm^-2^	nGy	nSv	cm^-2^
1	25	0.113	4.958E-06	4.265E-06	4.388E-06
2	60	0.094	4.719E-07	8.165E-07	8.847E-07
3	90	0.088	3.335E-07	5.367E-07	6.197E-07
4	120	0.089	3.362E-07	5.569E-07	5.627E-07
5	220	0.093	4.300E-07	5.900E-07	6.208E-07
6	400	0.114	6.929E-07	8.342E-07	8.658E-07
7	660	0.143	1.001E-06	1.173E-06	1.103E-06
8	1250	0.172	1.017E-06	1.162E-06	1.208E-06
9	3000	0.188	8.570E-07	9.533E-07	9.257E-07
10	6000	0.186	6.601E-07	7.242E-07	7.266E-07
11	8000	0.181	6.039E-07	6.621E-07	6.683E-07
12	10 000	0.172	5.505E-07	6.158E-07	6.080E-07
13	14 000	0.158	5.212E-07	5.674E-07	5.123E-07
14	20 000	0.138	6.531E-07	6.907E-07	7.255E-07

**Table 12:** Conversion coefficients valid for a detector with a cylindrical CeBr_3_ crystal of the dimensions 1.5″ × 1.5″ (diameter × length) and a thickness of the crystal casing of 5.0 mm).

		*w_i_* for conversion of counts to quantity …			
	*E*	Φ	*K*_a_	*H* ^*^(10)	*H*_p_ (10)
No.	keV	cm^-2^	nGy	nSv	nSv
1	25	0.8899	3.895E-05	3.351E-05	3.448E-05
2	60	0.1070	4.843E-07	8.881E-07	9.647E-07
3	90	0.0884	3.250E-07	5.508E-07	6.302E-07
4	120	0.0841	3.020E-07	5.130E-07	5.453E-07
5	220	0.0855	4.200E-07	5.800E-07	6.261E-07
6	400	0.1067	6.774E-07	8.355E-07	8.156E-07
7	660	0.1384	9.537E-07	1.142E-06	1.150E-06
8	1250	0.1652	1.040E-06	1.183E-06	1.197E-06
9	3000	0.1743	8.355E-07	9.353E-07	8.955E-07
10	6000	0.1620	6.162E-07	6.934E-07	6.864E-07
11	8000	0.1538	5.593E-07	6.259E-07	6.265E-07
12	10 000	0.1489	5.107E-07	5.690E-07	5.854E-07
13	14 000	0.1382	5.054E-07	5.674E-07	4.873E-07
14	20 000	0.1260	6.183E-07	6.907E-07	7.002E-07
